# PlyCYU endolysin targeting *Streptococcus agalactiae* exhibits a CHAP activity and a glucosaminidase domain mediating multimerization

**DOI:** 10.1128/aem.01872-24

**Published:** 2025-08-19

**Authors:** Sakunrat Ubonprasert, Wachiraporn Wachiradusit, Wichai Pornthanakasem, Warangkhana Songsungthong, Aritsara Jaruwat, Sasina Premjaichon, Tanaporn Uengwetwanit, Rinrada Suntivich, Konrawee Thananon, Kanyarat Suksomjaisaman, Jeerus Sucharitakul, Chutathip Puyprom, Tamonwan Lotangchanintra, Kanokwan Salamteh, Kittikhun Wangkanont, Channarong Rodkhum, Wonnop Visessanguan, Pimchai Chaiyen, Penchit Chitnumsub, Ubolsree Leartsakulpanich

**Affiliations:** 1 National Center for Genetic Engineering and Biotechnology (BIOTEC), National Science and Technology Development Agency (NSTDA) 67960, Khlong Luang, Pathum Thani, Thailand; 2 School of Biomolecular Science and Engineering, Vidyasirimedhi Institute of Science and Technology (VISTEC) 423058, Wang Chan District, Rayong, Thailand; 3 Department of Biochemistry, Faculty of Dentistry, Chulalongkorn University 543911, Bangkok, Thailand; 4 Center of Excellence for Molecular Biology and Genomics of Shrimp, Department of Biochemistry, Faculty of Science, Chulalongkorn University 543911, Bangkok, Thailand; 5 Center of Excellence in Molecular Crop, Department of Biochemistry, Faculty of Science, Chulalongkorn University 543911, Bangkok, Thailand; 6 Center of Excellence in Fish Infectious Diseases (CE FID), Faculty of Veterinary Science, Chulalongkorn University 26683, Bangkok, Thailand; 7 Siriraj Metabolomics and Phenomics Center, Faculty of Medicine Siriraj Hospital, Mahidol University 26685, Bangkok, Thailand; 8 Department of Biochemistry, Faculty of Medicine Siriraj Hospital, Mahidol University 549274, Bangkok, Thailand; INRS Armand-Frappier Sante Biotechnologie Research Centre, Laval, Quebec, Canada

**Keywords:** antibiotic alternative, amidase_5, CHAP domain, endolysin, *Streptococcus agalactiae*

## Abstract

Bacteriophage endolysins are attractive alternatives to antibiotics owing to their rapid action, host specificity, and unlikeliness of resistance development. Here, bioinformatic analysis of *Streptococcus suis* prophage sequences identified an endolysin, named PlyCYU, containing two putative catalytic domains—an N-terminal amidase_5 and a C-terminal glucosaminidase (Lyz2) domain—with two CW_7 family cell wall binding motifs. PlyCYU exhibited bactericidal activity against *Streptococcus agalactiae*, *Streptococcus dysgalactiae*, and *Streptococcus uberis,* with a minimum bactericidal concentration (MBC) range of 1.25 µM–40 µM, and retained bactericidal activity against *S. agalactiae* serotype II in ultra-high-temperature-processed milk (MBC 2.5 µM). Site-directed mutagenesis indicated that the amidase_5 domain was catalytically active and exhibited a cysteine-, histidine-dependent amidohydrolase/peptidase (CHAP) activity with the catalytic residues Cys34 and His99. Subdomain truncation analysis showed that PlyCYU214 and PlyCYU277, comprising the CHAP domain with one and two CW_7 motifs, respectively, conferred bactericidal activity, but lower than that of PlyCYU, while cyuLyz2 alone showed no activity. Notably, the bacteriolytic activity of PlyCYU277 was enhanced when cyuLyz2 was present. Agreeably, reducing sugars were detected in *S. agalactiae* lysis by PlyCYU and PlyCYU277 combined with cyuLyz2, but not by CHAP-inactive variants (PlyCYU-Cys34Ala/Ser and PlyCYU-His99Ala), PlyCYU277, and cyuLyz2 alone. This implied that cyuLyz2 action is CHAP dependent. Size exclusion chromatography (SEC) coupled with multi-angle light scattering and SEC-UV revealed PlyCYU and cyuLyz2 are homomultimers, whereas PlyCYU214 and PlyCYU277 are monomers. Therefore, the cyuLyz2 domain is important for the quaternary structure and the maximal activity of PlyCYU. Altogether, this study established PlyCYU endolysin as a potential antibiotic alternative against *Streptococcus*.

**IMPORTANCE:**

*Streptococcus agalactiae* is a major pathogen responsible for severe neonatal infections, bovine mastitis, and streptococcosis in fish. The increasing prevalence of multidrug-resistant bacteria presses the urgent need to discover antibiotic alternatives. Bacteriophage-derived endolysins represent a promising solution due to their ability to specifically and rapidly kill target bacteria and be less likely to develop resistance. Here, we identified and characterized a novel endolysin, PlyCYU, with potent bactericidal activity against different *Streptococcus* species, including *S. agalactiae*, *S. dysgalactiae*, and *S. uberis*, isolated from bovine and fish sources. This study also demonstrated the relationships between the structure assembly and activity of PlyCYU. PlyCYU forms a multimer, facilitated by its glucosaminidase (cyuLyz2) domain, for maximal activity. Altogether, we revealed that PlyCYU is a promising candidate for development as an antibiotic alternative for *Streptococcus* infection treatment and food safety applications, as well as for advancing our understanding of endolysin.

## INTRODUCTION

*Streptococcus agalactiae* or Group B *Streptococcus*, a gram-positive bacterium, is a major cause of neonatal sepsis in humans, bovine mastitis, and streptococcosis in farmed fish (1–4). *S. agalactiae* can produce a variety of virulence factors, including adhesins, capsules, hemolysins, enzymes, and biofilm (5–7). These play important roles in its pathogenicity, enabling the pathogen to evade the host immune system and complicating the treatment, posing a challenge in both human and animal infections. Antibiotics are conventionally used to treat bacterial infections, but the emergence of antibiotic resistance worldwide as the result of improper and excessive use in both the public health and agro-industry sectors has led to their ineffectiveness for treatment (8). *S. agalactiae* with varying susceptibilities to different antibiotics, as well as carrying antimicrobial resistance genes, has been increasingly reported in many regions of the world (9–14). Therefore, alternatives to antibiotics are desirable.

Endolysins are bacteriophage-encoded enzymes that mediate bacterial cell lysis and are required for the release of phage progenies during the lytic life cycle (15). Recombinant endolysins applied externally can effectively lyse and kill target bacteria (16). Due to their rapid killing kinetics, high specificity to target bacteria, activity against multidrug-resistant bacteria, and low tendency to induce resistance, endolysins constitute a promising class of antibiotic alternatives (17). Endolysins from bacteriophages targeting gram-positive bacteria have a characteristic modular structure consisting of at least two domains, including the enzymatic active domain (EAD), which digests the cell wall peptidoglycans, and a cell wall binding domain (CWD), which recognizes specific bacterial cell wall components. The EAD activities include glucosaminidase or muramidase, amidase, and peptidase, which cleave the β-1,4-glycosidic, MurNAc-L-Ala amide, and peptide bonds, respectively (18, 19). Several different endolysins targeting *S. agalactiae* have been reported, namely B30, PlyGBS, LambdaSa1, LambdaSa2, PlySK1249, PlySs1, PlySs2, and PlySs9 (16, 20–28). These endolysins contain diverse EADs and CWDs in various domain organizations, which contribute to different biochemical properties. In addition, recently discovered endolysins against streptococcal bacteria, which could be further developed for mastitis treatment, include Ply0643, which significantly reduces *S. agalactiae* in infected murine mammary glands (29), and EN534, which exhibits enhanced activity against *S. agalactiae* in the presence of Ca^2+^ ion (30). Characterizing new endolysins is important as they can provide endolysins with diverse mechanisms of action and stabilities, which can be combined to synergize the bacteriolytic efficacy. This synergy can be achieved by either combining endolysins that target different bonds in the peptidoglycan or combining endolysins and antibiotics that also target the bacterial cell wall. The use of endolysin combination can reduce the likelihood of antibiotic resistance, which is a crisis-emerging problem worldwide. Furthermore, the availability of a wide range of endolysins supplies a library of functional domains for engineering novel chimeric endolysins with enhanced or novel properties (31).

Here, we identified PlyCYU, a putative novel endolysin from a *Streptococcus suis* prophage named λSa2%2C. The search on *S. suis* genome was carried out since other studies have reported putative endolysins from *S. suis* genomes with activity against different streptococci and staphylococci. For example, PlySs2 and PlySs9 endolysins identified from *S. suis* prophage genomes have antibacterial activity against *Streptococcus uberis*, the causative agent of bovine mastitis (28). Furthermore, PlySs2 (CF-301) has been developed as an anti-*Staphylococcus* agent (32, 33). We expressed and evaluated bactericidal activity of PlyCYU against several strains of *S. agalactiae*, other *Streptococcus* spp., and *Staphylococcus aureus*. PlyCYU biochemical properties and the role of the two putative catalytic domains were characterized. We demonstrated for the first time that PlyCYU contains an N-terminal amidase_5 exhibiting cysteine-, histidine-dependent amidohydrolase/peptidase (CHAP) activity and a C-terminal glucosaminidase (cyuLyz2), whose activity requires prerequisite CHAP action and whose structure is required for domain-domain assembly in PlyCYU multimerization. Furthermore, the amidase_5 domain alone has a limited antibacterial activity in comparison with the full-length PlyCYU, suggesting that multimerization is important for maximizing the activity.

## RESULTS AND DISCUSSION

### Sequence and structure analysis

**Fig 1:**
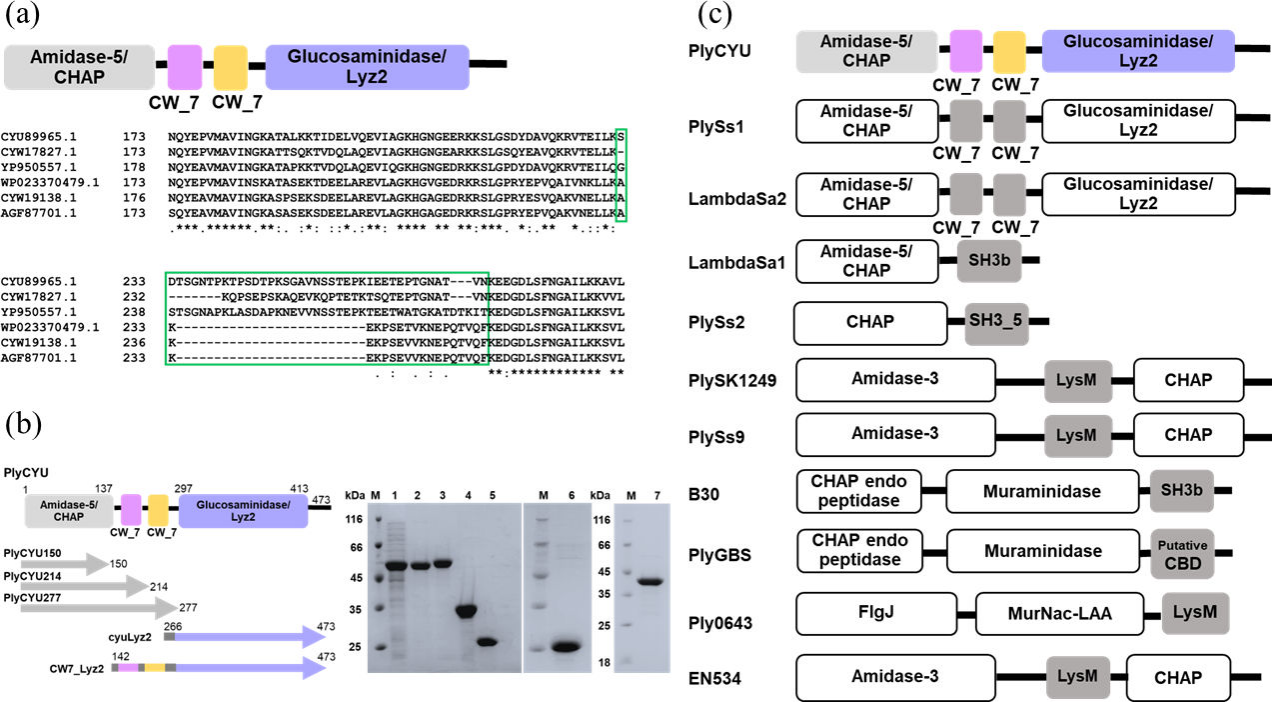
PlyCYU and its variants. (a) Schematics of modular organization and multiple sequence alignments of endolysins in this study—CYU89965.1, CYW17827.1, YP950557.1, WP023370479.1, CYW19138.1, and AGF87701.1—showing N-terminal amidase_5 domain, and varying linker lengths tethering the second cpl-7 motif and the C-terminal glucosaminidase domain, either short (18 amino acids) or long (32–43 amino acids) sequences as indicated in green boxes. (b) Schematics showing PlyCYU full-length and domain-truncated constructs and their subunit molecular weights based on SDS-PAGE (12%) analysis. Samples of crude extract of tag-free PlyCYU, purified tag-free PlyCYU (51.8 kDa), His_6_-tagged PlyCYU (53.9 kDa), PlyCYU277 (32.6 kDa), PlyCYU214 (26.0 kDa), cyuLyz2 (24.9 kDa), and CW7_Lyz2 (37.9 kDa) were in lanes 1–7, respectively. Lanes marked M represent protein marker (Pierce unstained protein MW marker). (c) Modular organization of PlyCYU and other endolysins targeting *S. agalactiae*.

We performed a search on NCBI for endolysins in *S. suis* genome and annotated protein sequences by Conserved Domain Database (CDD) search. We identified eight putative endolysins carrying two catalytic domains, consisting of a putative N-terminal amidase_5 (CHAP) and C-terminal glucosaminidase (Lyz2; lysozyme subfamily 2) (Fig. 1a). In addition, two putative cpl-7 cell wall binding motifs belonging to CW_7 superfamily were identified in between the two catalytic domains. The sequence alignments revealed that the other sequences share 68%–88% identity to CYU89965.1 (Fig. S1). The linker lengths tethering the second cpl-7 motif and the C-terminal glucosaminidase domain are either short (18 amino acids) or long (32–43 amino acids) (Fig. 1a; Fig. S1), which may modulate antibacterial activity of endolysins as noted previously (34). Therefore, three sequences—WP023370479.1 for the short linker (18 amino acids) and CYU89965.1 and YP950557.1 for a long linker (40 and 43 amino acids)—were expressed and purified. However, only CYU89965.1 (PlyCYU) was studied extensively, as the others showed poor or no binding affinity to the Ni-Sepharose column despite several attempts of purification.

The modular structure of PlyCYU is organized from the N-terminal amidase_5 (residues 4–137), followed by two putative cpl-7 cell wall binding motifs (residues 149–187 and 193–231), then the C-terminal glucosaminidase domain (residues 297–413) (Fig. 1b). Notably, CDD search predicts glucosaminidase domain with a relatively low confident E-value of 3.6e − 03, compared to the higher confident E-values of amidase_5 (2.2e − 65) and cpl-7 (3.0e − 14 and 4.5e − 09), respectively. Phylogenetic analysis of PlyCYU with other 10 reported endolysins against *S. agalactiae* reveals that PlyCYU is grouped with PlySs1 endolysin from *S. suis* phage and LambdaSa2 from *S. agalactiae* phage (Fig. S2a). Like PlyCYU, PlySs1 and LambdaSa2 are annotated to contain amidase_5 and glucosaminidase domains with an intervening dual cpl-7 motifs, sharing 77% and 74% sequence identity, respectively (Fig. 1c; Fig. S2b) (22, 23, 25). On the other hand, LambdaSa1 from *S. agalactiae* phage carries only one amidase_5 catalytic domain followed by an SH3b cell wall binding domain, sharing 40% sequence identity to PlyCYU (Fig. 1c; Fig. S2b). The amidase_5 of LambdaSa1 and LambdaSa2 possesses γ-D-glutaminyl-L-lysine endopeptidase activity (22). The other seven endolysins—PlySs2, PlySK1249, PlySs9, B30, PlyGBS, EN534, and Ply0643—possess modular structures with distinct catalytic and cell wall binding domains (24, 29, 30, 35), sharing only 10%–13% sequence identity with PlyCYU (Fig. 1c; Fig. S2c). Given that PlyCYU endolysin contains two different putative EADs, despite the glucosaminidase domain being predicted with a low E-value, it was hypothesized that their functions may cooperate to synergistically enhance the bacteriolytic activity.

### Biochemical characterizations of recombinant PlyCYU

**Fig 2:**
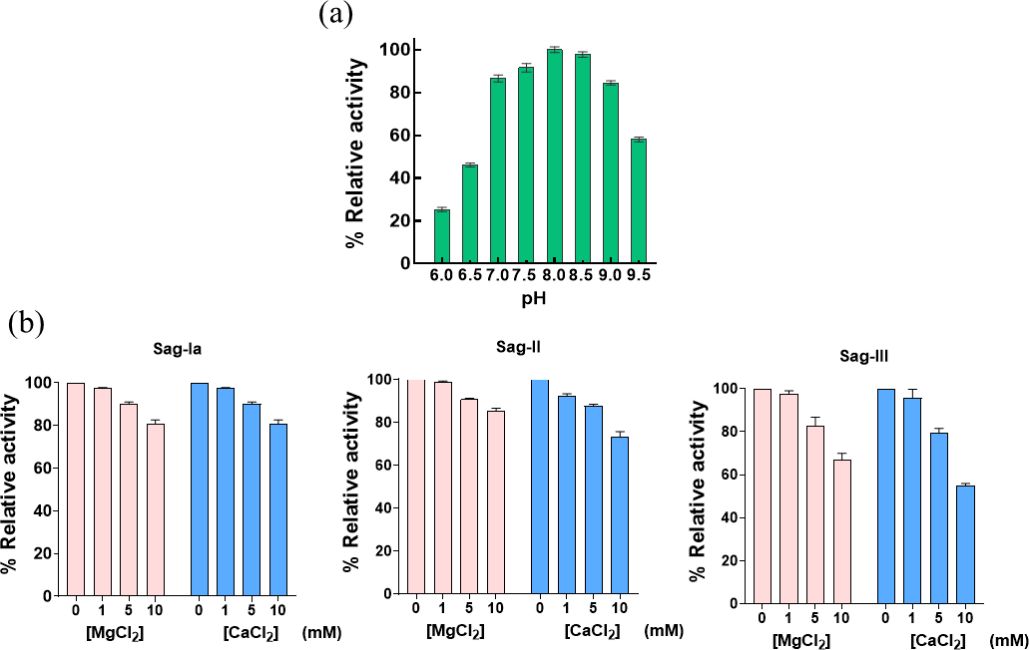
Effects of pH and divalent metals on PlyCYU activity. (a) pH activity profile of PlyCYU in poly-buffers pH 6.0–9.5 against *S. agalactiae* serotype II. (b) Effects of Ca^2+^ and Mg^2+^ ions on PlyCYU activity. Plots showing percent relative activity of PlyCYU (2 µM) in the presence of CaCl_2_ and MgCl_2_ (0 mM–10 mM) against *S. agalactiae* serotypes Ia, II, and III. The data shown are average values with error bars representing standard deviation from triplicate experiments.

The recombinant PlyCYU was heterologously expressed as a soluble protein with a molecular mass of ~54 kDa (Fig. 1b), in agreement with the calculated molecular mass. To assess the activity and the pH optimum of PlyCYU, the bacteriolytic activities of PlyCYU against *S. agalactiae* serotype II were determined at various pH using turbidity reduction assay. PlyCYU exhibited ≥85% bacteriolytic activity over a pH of 7.0–9.0, with optimum activity at pH 8.0 (Fig. 2a). Of note, 58 and 46% of activities were achieved at pH 9.5 and 6.5, respectively; however, the activity of PlyCYU was significantly diminished at a lower pH.

To explore the possibility of using PlyCYU in milk-related applications, the effects of divalent metals Mg^2+^ and Ca^2+^ were explored against *S. agalactiae* serotypes Ia, II, and III as these metals are main elements present in milk. The activity of PlyCYU was highest without the addition of Mg^2+^ and Ca^2+^. The presence of Mg^2+^ or Ca^2+^ reduced PlyCYU bacteriolytic activity in a dose-dependent manner for all three serotypes (Fig. 2b). PlyCYU activity was more susceptible to Ca^2+^ than Mg^2+^ at 10 mM. *S. agalactiae* serotype III was the most susceptible among the three serotypes to inhibition by Mg^2+^ and Ca^2+^.

As PlyCYU confers bacteriolytic activity against *S. agalactiae* over a pH range of 7.0–9.0, its use can be applied in both mild acid and alkaline conditions. In bovine mastitis, *S. agalactiae* infection causes an increase of the milk pH from 6.65 ± 0.01 in healthy cows to 7.10 ± 0.01 in subclinical mastitis (36), where PlyCYU exhibits 87% activity (Fig. 2a). Given the concentrations of free Ca^2+^ and Mg^2+^ in cow milk of 2.0 and 0.8 mM, respectively (37–39), our results suggest that PlyCYU has a potential to further develop as a new alternative to antibiotics to treat bovine mastitis caused by *S. agalactiae* infection.

PlyCYU activity covers a pH range similar to that of PlySs1 and LambdaSa2, with the latter having a broader pH range (5.5–9.5) (16, 22, 25). On the other hand, B30 displays the optimal activity at pH 5.5–6.0, which is less than the pH in milk milieu of bovine mastitis and also requires 10 mM CaCl_2_ for optimal activity (16, 24).

To determine the thermodynamic stability of PlyCYU endolysin, a thermofluor assay was performed in various buffers and pH conditions. The assay measures the temperature inducing protein denaturation by following fluorescence change of an environmentally sensitive fluorescent dye, such as SYPRO orange (40). The melting temperature (T_m_) values indicated that pH affected the PlyCYU stability (Table 1). We observed that PlyCYU had the lowest T_m_ at pH 5, which increased with rising pH and reached a maximum at pH 6–7.5 in the presence of 50 mM–150 mM NaCl, but 300 mM NaCl lowered T_m_ by ~1°C. Therefore, PlyCYU has been stored in 50 mM HEPES, pH 7.5, 50 mM NaCl, and 20% glycerol to avoid protein aggregation due to its pI value (6.79). According to the T_m_ values, PlyCYU was stable over the pH range 6–9, which is in good agreement with PlyCYU pH activity profile that PlyCYU was optimally active at pH 7–9 at 37°C.

**TABLE 1:** T_m_ of PlyCYU endolysin in various buffers and pHs with 50, 150, and 300 mM NaCl in the presence of 20% glycerol

Buffer	pH	T_m_ (°C)		
		50 mM NaCl	150 mM NaCl	300 mM NaCl
Acetate	5	35.7 ± 0.6	32.0 ± 0.0	30.5 ± 0.6
MES	6	45.5 ± 0.4	45.8 ± 0.2	45.2 ± 0.2
Potassium phosphate	7	45.6 ± 0.2	45.6 ± 0.1	45.0 ± 0.2
HEPES	7	46.5 ± 0.1	45.7 ± 0.1	44.9 ± 0.0
HEPES	7.5	46.1 ± 0.2	45.6 ± 0.3	44.8 ± 0.2
Tris-HCl	8	45.2 ± 0.1	44.8 ± 0.1	44.1 ± 0.1
Tris-HCl	8.5	45.2 ± 0.1	44.7 ± 0.2	43.9 ± 0.2
Tris-HCl	9	42.9 ± 0.6	43.2 ± 0.2	41.7 ± 0.5

### Specificity of PlyCYU bactericidal activity

Bactericidal activity of PlyCYU was determined against various bacterial species and strains, including *S. agalactiae* (serotypes Ia, II, and III, and isolates from bovine [*Bos taurus*], tilapia [*Oreochromis niloticus*], and snakeskin gourami [*Trichopodus pectoralis*]), *Streptococcus dysgalactiae*, *S. uberis*, and *Staphylococcus aureus*, and methicillin-resistant *S. aureus* (MRSA) (Table 2). To assess the activity and specificity of PlyCYU, we determined the minimum bactericidal concentration (MBC) against each bacterial species/strain/isolate (Table 2). PlyCYU exhibited bactericidal activity against all *S. agalactiae* strains tested with the MBCs ranging from 1.25 to 40 µM. In addition, PlyCYU killed other *Streptococcus* species at comparable MBC to *S. agalactiae* (1.25 µM for *S. dysgalactiae* and 5 µM for *S. uberis*). By contrast, its activity against *S. aureus* and MRSA strains was significantly lower (MBC >320 µM), indicating a higher specificity for *Streptococcus* spp.

**TABLE 2:** Antibacterial activity (MBC) of PlyCYU against different bacteria^*a*^

Name in this study	MBC (μM)	Bacterial information
Sag Ia	10	*S. agalactiae* ATCC BAA-1138, serotype Ia
Sag II	1.25	*S. agalactiae* ATCC BAA-1175, serotype II
Sag III	40	*S. agalactiae* ATCC BAA-22-MINI-PACK, serotype III
Sag34	1.25	*S. agalactiae* ATCC 27956, bovine udder infection
Sag35	2.5	*S. agalactiae* ATCC 7077, mastitis cow milk
Sag36	1.25	*S. agalactiae* ATCC 12928, bovine mastitis
Sag37	1.25	*S. agalactiae* ATCC 49448, bovine milk
Sag41	5	*S. agalactiae* ATCC 27541, bovine mammary gland
Sag CU02	20	*S. agalactiae* CU02, tilapia
Sag CU05	20	*S. agalactiae* CU05, tilapia
Sag RTCR2003	10	*S. agalactiae* RTCR2003, tilapia
Sag R2	5	*S. agalactiae* R2, snakeskin gourami
Sag R5	1.25	*S. agalactiae* R5, snakeskin gourami
*S. dysgalactiae*	1.25	*S. dysgalactiae* ATCC 43078, Group C, cow
*S. uberis*	5	*S. uberis* ATCC 19436, Type I
*S. aureus*	320	*S. aureus* DMST 4745
MRSA 7	>320 (98%)	*S. aureus* MRSA-7 (BEI NR-13530)
MRSA 131	>320 (99%)	*S. aureus* MRSA-131 (BEI HM-466)
MRSA M0001	>320 (99%)	*S. aureus* MRSA-M0001 (BEI NR-41875)
MRSA S0385	>320 (99%)	*S. aureus* MRSA-S0385 (BEI NR-28983)

^*a*^ % Bacterial reduction (cfu/mL) of MRSA at 0.32 mM PlyCYU treatment.

A possible barrier to the use of endolysin for treating bovine mastitis is the binding of endolysin to fat in milk, which interferes with the binding of endolysin to bacteria and the bactericidal activity (41). Therefore, we determined the antibacterial activity of PlyCYU against *S. agalactiae* suspended in UHT (ultra-high-temperature-processed) milk to assess its potential as an anti-mastitis agent. PlyCYU demonstrated an MBC value of 5 µM after 1 and 3 h treatment and 2.5 µM after 6 h treatment, comparable to that obtained from a standard assay in cation-adjusted Mueller–Hinton broth (CAMBH) supplemented with 25% (vol/vol) horse serum (Table 3). However, the reduction *S. agalactiae* level (cfu/mL) in the standard medium was 0.11–0.28 log_10_ higher than those in UHT milk. Nonetheless, the observed activity of PlyCYU in UHT milk suggests its potential as an antibiotic alternative for the treatment of bovine mastitis. A number of endolysins with anti-*S*. *agalactiae* activity in UHT milk have been reported, including LambdaSa2 and NC5 (31, 42). Their activities cannot be directly compared due to varied reagents and conditions used such as bacterial strains, bacterial inoculum number, and incubation time. A 3-log bacterial reduction obtained here is biologically meaningful as this reduces bacterial numbers by 1,000-fold. The MBC value can be a guide for a usage dose in application. It should be mentioned that UHT milk cannot completely represent the raw milk; therefore, the lytic activity of endolysins acquired from these milks may vary as described previously (31, 43). Evaluating PlyCYU efficacy through mammary infusion in a mouse model could further demonstrate its potential as an alternative agent for mastitis control.

**TABLE 3:** Antibacterial activity of PlyCYU against *S. agalactiae* serotype II resuspended in CAMHB supplemented with 25% (vol/vol) horse serum (HS) and UHT milk^*a*^

Treatment time (h)	MBC of PlyCYU (µM)		Log_10_ bacterial reduction (cfu/mL)		*P*-value
	CAMHB+ 25% HS	UHT milk	CAMHB+ 25% HS	UHT milk	
1	5	5	3.14 ± 0.02	3.03 ± 0.04	0.026
3	5	5	3.32 ± 0.07	3.06 ± 0.09	0.034
6	2.5	2.5	3.31 ± 0.01	3.03 ± 0.04	0.001

^*a*^ The log_10_ bacterial reduction values shown are the average ± SD of triplicate experiments. The two-tailed unpaired *t*-test was performed, and the *P*-value ≤0.05 was considered statistically significant.

### Time-kill kinetics of PlyCYU bactericidal activity against *S*. *agalactiae*

**Fig 3:**
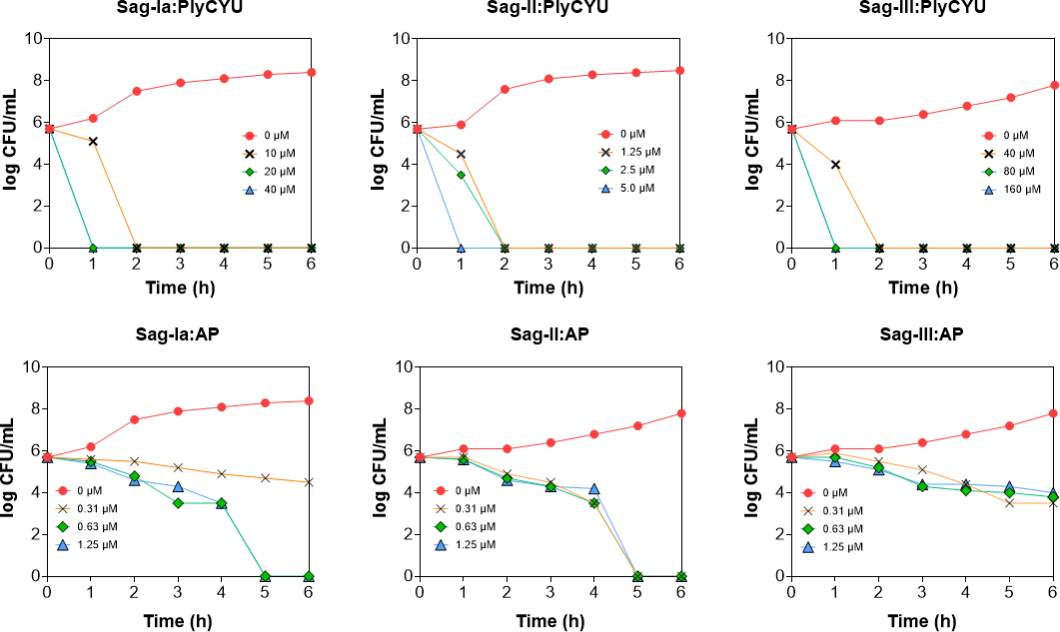
Time-kill kinetic curves of PlyCYU and AP at 1×, 2×, and 4× MBC against *S. agalactiae* serotypes Ia, II, and III.

The time-kill kinetics of PlyCYU bactericidal activity was compared with those of ampicillin (AP), which similarly acts at the bacterial cell wall. PlyCYU hydrolyzes peptidoglycan components in the cell wall, whereas AP inhibits cell wall transpeptidase. Both actions lead to cell lysis. At concentrations corresponding to 1–4× MBC against three *S*. a*galactiae* serotypes, PlyCYU at 1× MBC effectively cleared 10^6^ cfu/mL *S*. *agalactiae* serotypes Ia, II, and III within 2 h and complete lysis was achieved in 1 h at PlyCYU ≥2× MBC (Fig. 3). On the other hand, AP at 1× MBC (0.31 µM) against serotypes Ia, II, and III barely lysed *S. agalactiae* in 2 h (Fig. 3). Complete lysis of *S. agalactiae* serotype Ia by AP was achieved at 2–4× MBC in 5 h, that of serotype II was observed at 1–4× MBC within 5 h, while 4× MBC reduced serotype III by 2 log_10_ cfu/mL in 6 h (Fig. 3). In summary, the time-kill kinetics results indicated that PlyCYU action was relatively rapid (compared to AP), a distinguishing characteristic of endolysins, as they directly lyse peptidoglycans thereby causing rapid cell lysis and death.

### Effects of PlyCYU on bacterial cell morphology

**Fig 4:**
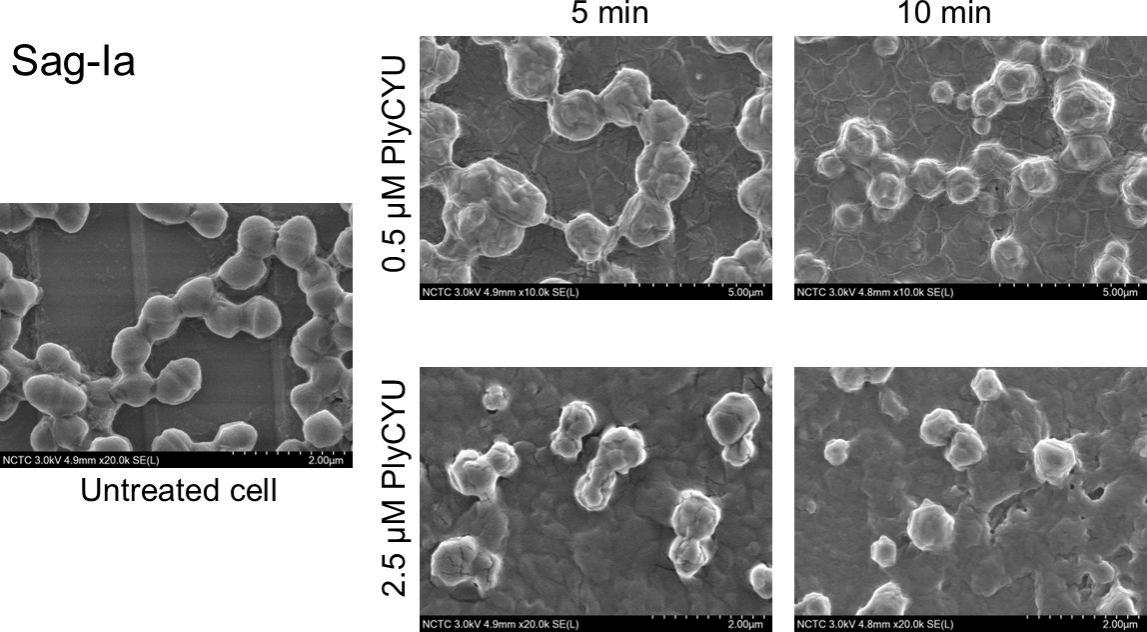
Scanning electron micrographs of untreated *S. agalactiae* serotype Ia and those treated with PlyCYU.

Because endolysin is a class of peptidoglycan-cleaving enzymes, cell wall damage is expected upon treatment. Bacteria cell morphologies of *S. agalactiae* serotype Ia were visualized by scanning electron microscopy (SEM) after treatment with 0.5 and 2.5 µM PlyCYU for 5 and 10 min. Untreated *S. agalactiae* serotype Ia displayed the standard long chains of oval-shaped bacteria with a smooth surface (Fig. 4), while upon exposure to PlyCYU, the cells displayed a de-chaining feature with an irregular cell deformation and shriveled surface; cell debris could be observed in the background (Fig. 4). The results here confirmed the antibacterial activity of PlyCYU against *S. agalactiae* as shown in the above section.

### Identification of PlyCYU catalytic residues

**Fig 5:**
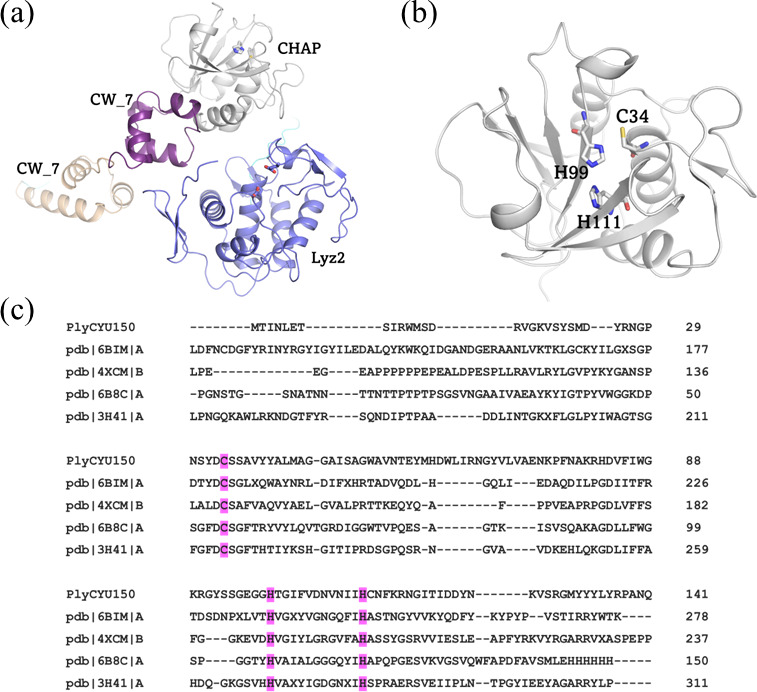
Modular structure of PlyCYU endolysin and functional analysis of the CHAP domain. (a) Monomeric structure of the full-length PlyCYU predicted by AlphaFold2, composed of two putative catalytic domains (CHAP and Lyz2 glucosaminidase domains), and two cell wall binding motifs (CW_7). The models for the CHAP and Lyz2 glucosaminidase domains were predicted with very high confidence (pLDDT > 90) combined with some small parts in 90 > pLDDT > 70. (b) CHAP (residues 1–141) shows a catalytic triad of residues Cys34, His99, and His111. (c) Sequence alignment of the CHAP domain (PlyCYU150) of PlyCYU with other known structures of peptidoglycan hydrolases in the PDB (code: 6BIM, 4XCM, 6B8C and 3H41) highlighting the catalytic triad of cysteine and two histidines in pink.

Due to low sequence identity of PlyCYU with other proteins in the Protein Data Bank (PDB), a protein similarity search was conducted by NCBI Blast with the AlphaFold database (https://www.ebi.ac.uk/Tools/sss/ncbiblast) (44, 45) to obtain a 3D model of PlyCYU. *S. suis* glucosaminidase domain-containing protein (473 amino acids) (AF-A0A0Z8G0W0-F1) with 100% sequence identity to PlyCYU was the top rank model. The predicted PlyCYU structure comprises four independent domains (Fig. 5a), consistent with the results obtained from the CDD search. The N-terminal domain of PlyCYU (residues 1–141) displayed a common core of N1pC/P60 cysteine peptidase (CHAP) superfamily, with a catalytic triad of one cysteine and two histidines (Fig. 5b). Structure alignment using DALI (http://ekhidna2.biocenter.helsinki.fi/dali/) (46) of the CHAP domain of PlyCYU with related protein structures in PDB also identified the conserved Cys/His/His catalytic triad despite overall low sequence identities (Fig. 5c). To confirm the necessity of the key catalytic triad residues for peptidoglycan cleavage activity, we generated PlyCYU-Cys34Ala/Ser and PlyCYU-His99Ala mutants, which were successfully expressed as recombinant soluble proteins, indicating that these mutations did not perturb the overall protein structure. These PlyCYU variants could not lyse *S. agalactiae* cells (Fig, S3), and they lacked bactericidal activity against *S. agalactiae* serotypes tested even at 100 µM protein (Table S1). This result suggested that the glucosaminidase domain may not inherit bactericidal activity. Together, the findings not only highlighted the role of the catalytic triad of CHAP but also suggested that CHAP activity is sufficient for PlyCYU bacteriolytic activity.

### Characterization of PlyCYU truncated variants

To test the notion if PlyCYU bacteriolytic activity was dependent on CHAP, we generated three truncated variants of the CHAP−PlyCYU150 (amino acids 1−150, containing only the amidase_5 domain), PlyCYU214 (amino acids 1−214, containing an additional cpl-7 motif), and PlyCYU277 (amino acids 1−277, containing additional two cpl-7 motifs). In addition, cyuLyz2 (amino acids 266 to 473, containing only the glucosaminidase domain) and CW7_Lyz2 (amino acids 142 to 473, containing two cpl-7 motifs and the glucosaminidase domain) were constructed to explore the role of glucosaminidase domain in the lytic activity of the full-length PlyCYU (Fig. 1b). The latter was included because the absence of cpl-7 may influence the glucosaminidase activity due to the lack of CWD. PlyCYU150 was not tested for function due to its high propensity to aggregate. The antibacterial activity of each variant was determined against three *S*. *agalactiae* serotypes using a viable cell count assay. MBC values of PlyCYU214 for *S. agalactiae* serotypes Ia (20 µM) and II (5 µM) were double of the intact PlyCYU, and those of PlyCYU277 for both serotypes Ia and II (10 µM) were equal to PlyCYU’s MBC for serotype Ia (Table 4). However, at 80 µM, the maximum concentration tested, PlyCYU214 and PlyCYU277 reduced the level of *S. agalactiae* III by 99.83 and 99.86 %, respectively, not reaching the MBC. The cyuLyz2 and CW7_Lyz2 constructs for the glucosaminidase domain did not show bactericidal activity (Table 4), in agreement with the above studies using CHAP catalytic mutants. Taken together, the dissected PlyCYU variants showed lower activity than the full-length PlyCYU, implying that the incorporation of glucosaminidase domain is important for maximal activity.

**TABLE 4:** Antibacterial activity and molecular masses of PlyCYU variants^*a*^^,^^*b*^

Construct	MBC (µM)			Subunit molecular mass (kDa)	Molecular mass (kDa) (calculated oligomeric number)	
	Sag Ia	Sag II	Sag III		SEC-MALS^*c*^	SEC-UV
PlyCYU	10	2.5	80	53.9	278.4 (5.2)	368 (6.8)
PlyCYU214	20	5	>80 (99%)	26.0	–	8.0 (0.3)
PlyCYU277	10	10	>80 (99%)	32.6	44.4 (1.4)	28.0 (0.9)
cyuLyz2	na	na	na	24.9	97.2 (3.9)	176 (6.9)
CW7_Lyz2	na	na	na	37.9	166.3 (4.4)	447 (11.8)

^*a*^ na, no detectable antibacterial activity; –, not performed.

^*b*^ % Bacterial reduction (cfu/mL) at 80 µM treatment.

^*c*^ SEC-MALS, size exclusion chromatography coupled with multi-angle light scattering.

**Fig 6:**
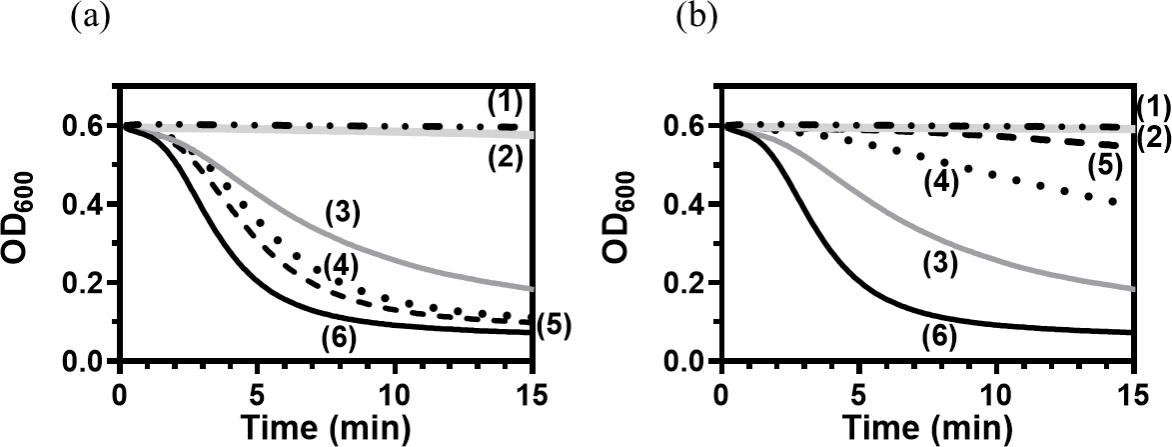
Bacteriolytic activities of PlyCYU variants and their combination using turbidity reduction assay. (a) Endolysins included were (1) none (*S. agalactiae* serotype II control; dash and dot line), (2) cyuLyz2 (50 µM; light gray), (3) PlyCYU277 (5 µM; gray), (4) PlyCYU277 and cyuLyz2 (1:1; 5 µM each; dot), (5) PlyCYU277 and cyuLyz2 (1:5; 5:25 µM; dash line), and (6) PlyCYU (5 µM; black). (b) The experiments were performed similar to (a) but CW7_Lyz2 was used, instead of cyuLyz2.

It is hypothesized that the lack of antibacterial activity observed by cyuLyz2 alone may be because its activity depends on the action of the CHAP domain. We next probed this possibility using a turbidity reduction assay. While PlyCYU rapidly dropped the cell turbidity, PlyCYU277 showed reduced activity, and both cyuLyz2 and CW7_Lyz2 alone showed no measurable cell turbidity change (Fig. 6). The results were consistent with those of the viable cell assay described above. However, the addition of cyuLyz2 to PlyCYU277 at a 1:1 ratio increased the lytic activity in comparison to that of PlyCYU277 alone. Increasing the molar ratio of cyuLyz2 (1:5) slightly enhanced the bacteriolytic effect but did not reach the level observed for PlyCYU (Fig. 6a). In contrast, the addition of CW7_Lyz2 to PlyCYU277 reduced lytic activity, and the effect was more pronounced at higher molar ratio of CW7_Lyz2 (1:5) (Fig. 6b). Notably, CW7_Lyz2 differs from cyuLyz2 by containing two CW_7 motifs. The presence of two CW_7 motifs in both PlyCYU277 and CW7_Lyz2 may competitively bind to the bacterial cell wall, thereby interfering with the proper function, but the underlying mechanism needs further exploration.

The observed activity for the combination of PlyCYU277 and cyuLyz2 prompted us to explore whether cyuLyz2 possesses inherent glycosidase activity using Park Johnson assay (47). The reducing sugar released by PlyCYU and its truncated variants was assessed. A reaction of *Micrococcus lysodeikticus* lysis by chicken egg white lysozyme (0.3 µM), as a positive control, released 9.2 ± 0.5 µM reducing sugar at 37°C for 30 min (Table S2). The bacteriolytic reaction of *S. agalactia*e serotype II by PlyCYU (10 µM) generated 10.3 ± 0.3 µM reducing sugar, whereas no sugar was detected for the reaction with CHAP inactive variants (PlyCYU-Cys34Ala and PlyCYU-His99Ala) or with truncated variants (PlyCYU277, cyuLyz2, and CW7_Lyz2) (Table S2). The mixture of PlyCYU277 with cyuLyz2 at equimolar ratio (20 µM each) yielded detectable sugar (3.1 ± 0.4 mM), but less than that derived from 10 µM PlyCYU reaction (10.3 ± 0.3 µM) (Table S2). Notably, reducing sugar was not detected for the reaction conducted with the mixture of PlyCYU277 and CW7_Lyz2. These results suggest that the cyuLyz2 domain possesses glycosidase activity, and the activity of the CHAP domain is vital for cyuLyz2 function.

### Molecular mass determination by size exclusion chromatography coupled with multi-angle light scattering (SEC-MALS) and traditional SEC-UV

To explore the role of each domain on PlyCYU quaternary structure, we analyzed the molecular mass of the PlyCYU and the truncated variants using SEC-MALS and traditional SEC-UV (Table 4). SEC-MALS determined the molecular masses and corresponding oligomeric states in solution of PlyCYU, cyuLyz2, and CW7_Lyz2 to be 278.4 for pentamer, 97.2 for tetramer, and 166.3 for tetramer, respectively (Table 4; Fig. S4I). PlyCYU277 is a monomer based on the molecular mass of 44.4 kDa (Table 4; Fig. S4I). Traditional SEC-UV determined the molecular masses of PlyCYU, cyuLyz2, CW7_Lyz2, PlyCYU277, and PlyCYU214 to be 368 (heptamer), 176 (heptamer), 447 (dodecamer), 28 (monomer), and 8 kDa (monomer), respectively (Table 4; Fig. S4II). The higher molecular masses found from traditional SEC-UV suggested that these PlyCYU variants possess non-globular shape. SEC-UV can overestimate size if a protein or oligomer has a more extended or non-globular shape (48). In contrast, PlyCYU214 showed very low molecular mass even when performing SEC in the presence of 500 mM NaCl to avoid the interaction with the column matrix, suggesting that its structure is very compact. Together, the SEC-MALS and SEC-UV suggested that PlyCYU, cyuLyz2, and CW7_Lyz2 can undergo self-assembly in solution, but not the PlyCYU277 and PlyCYU214. Despite differences in molecular mass values between techniques, both suggested that PlyCYU, cyuLyz2, and CW7_Lyz2 inherit self-assembly propensity. Therefore, we hypothesized that multimerization of PlyCYU involved domain-domain interactions of the glucosaminidase cyuLyz2. Further studies are needed to provide insight into self-assembly of PlyCYU and the relationship between PlyCYU oligomerization and its maximal activity.

Given that both PlySs1 and LambdaSa2 harbor a C-terminal glucosaminidase domain and they share >70% identity to PlyCYU, multimerization properties may be conserved in these endolysins, driven by glucosaminidase domain, to regulate the optimal activity. Further studies of these domains could provide insight into their mechanism of action and evolutionary conservation of endolysins. To date, the role of the bactericidal silent domain is not fully understood. However, the amidase_3 domain of LysSA12 and LysSA97 has been reported to be catalytically inactive but promotes CWD binding to the bacterial hosts for efficient bacteriolysis (49). The CHAP endopeptidase domain of PlySK1249 has a de-chaining activity that cooperates with the N-terminal amidase in bacteriolysis (26, 50).

Multimeric endolysins have been reported for several endolysins targeting gram-positive bacteria such as streptococcal phage PlyC endolysin (51) and enterococcal phage Lys170 endolysin (52). PlyC is an assembled endolysin composed of two protein components, PlyCA for catalytic activity and PlyCB for cell wall binding, encoded from two separated genes (51). The catalytically active PlyC is composed of one PlyCA subunit and eight PlyCB subunits (51). Lys170 is an assembly of one full-length and three subunits of co-eluted truncated C-terminal CWD, derived from an internal translation start site of the full-length gene of the Lys170 endolysin. Therefore, PlyC and Lys170 are heteromeric endolysins. PlyCYU is a homo-oligomer as the SDS-electrophoresis showed only a single full-length PlyCYU. Here, we show for the first time that the C-terminal CHAP-dependent cyuLyz2 domain is involved in the formation of PlyCYU multimer. Further studies are needed to understand how multimerization of PlyCYU is associated with enhanced bactericidal activity.

### Conclusion

Here, we identified a novel endolysin encoded by *S. suis* prophage and demonstrated the antibacterial activity against *S*. *agalactiae*, a bacterial pathogen causing various diseases in humans and animals. PlyCYU showed bacteriolytic activity over a pH range of 7.0–9.0 and a fast clearance of *S. agalactiae*. PlyCYU endolysin has two annotated EADs, but only the N-terminal amidase_5/CHAP domain, with a catalytic Cys/His/His triad, exhibits bactericidal activity that also requires the presence of two downstream cell wall binding cpl-7 motifs for optimal activity, as shown by PlyCYU214 and PlyCYU277. We report for the first time the role of the glucosaminidase cyuLyz2 domain in mediating the multimerization of PlyCYU. Future exploration of PlyCYU structure will allow elucidation of the functional relationships of these domains. A better understanding of the multi-modular architecture of endolysins should advance the rational design of endolysins in the world of antibiotic-resistant pathogenic microbials.

## MATERIALS AND METHODS

### Bioinformatics analysis

The putative endolysin sequences in *S. suis* were searched and analyzed for conserved domain using NCBI/CDD-based annotation (53). Phylogenetic analysis was performed by neighbor-joining method in Molecular Genetics Analysis version 11 (54, 55). Amino acid sequence alignments of PlyCYU with other endolysins were performed using ClustalW (https://www.genome.jp/tools-bin/clustalw). The structure of the PlyCYU endolysin was predicted by conducting a protein similarity search using NCBI Blast with the PDB and the AlphaFold database (https://www.ebi.ac.uk/Tools/sss/ncbiblast) (44, 45). Then, the predicted 3D structure of PlyCYU was aligned with other related proteins in the PDB using DALI (46) to identify conserved amino acids. Figures were generated using the PyMOL program (https://www.pymol.org).

### Bacterial strains and growth conditions

Bacterial strains used in this study are described in Table 2. *S. agalactiae*, *S. dysgalactiae*, *S. uberis*, and *S. aureus* were cultured on brain heart infusion (BHI) agar (Difco Laboratories) plates at 37°C with the air in the incubator regulated at 5% CO_2_.

### Cloning of PlyCYU endolysin variants

A putative endolysin (GenBank: CYU89965.1, 473 amino acids) from the λSa2%2C prophage of *S. suis*, designated here as PlyCYU, was selected from the NCBI database. The PlyCYU endolysin gene was codon-optimized and chemically synthesized for expression in *Escherichia coli* by GenScript. The PlyCYU gene was cloned into pET28a (Novagen) at the *Nco*I and *Not*I sites to generate a tag-free PlyCYU construct, and at the *Nde*I and *Not*I sites to generate an N-terminal His_6_-tagged PlyCYU.

The pET28a-His_6_-tagged-PlyCYU plasmid was used as a template for PCR amplification of genes encoding various truncated forms of His_6_-tagged PlyCYU using primer pairs listed in Table S3. Site-directed mutagenesis was performed to mutate Cys34 to Ala/Ser and His99 to Ala according to QuikChange site-directed mutagenesis protocol (Stratagene) and primer pairs listed in Table S3. All constructs were validated by DNA sequencing. The plasmids obtained were transformed into *E. coli* BL21(DE3) for protein expression.

The tag-free PlyCYU was used to demonstrate antibacterial activity of PlyCYU in terms of the minimum bactericidal concentration, time-kill kinetics, and cell morphology change. The His_6_-tagged version was used to characterize and compare biochemical properties of the full-length PlyCYU with the PlyCYU variants.

### Endolysin expression and purification

*E. coli* BL21(DE3) harboring each of the PlyCYU endolysin variant expression plasmids was grown in autoinduction media (56) composed of 0.5% wt/vol yeast extract, 1% wt/vol peptone, 1% wt/vol NaCl, 0.05% wt/vol glucose, 0.2% wt/vol lactose, 2 mM MgSO_4_, 5 mM Na_2_SO_4_, 1× NPS (50 mM NH_4_Cl, 25 mM KH_2_PO_4_, and 25 mM Na_2_HPO_4_), and 100 µg/mL kanamycin at 37°C for 3 h. Overexpression of the PlyCYU endolysin was induced at 20°C for 20 h. The cells were harvested by centrifugation at 8,000 × *g* for 10 min at 4°C and kept at −20°C until use.

The His_6_-tagged PlyCYU (53.9 kDa) was purified by Ni-Sepharose affinity chromatography. Briefly, cells resuspended in 20 mM imidazole (Im) in buffer A (100 mM phosphate buffer, pH 7.5, containing 400 mM NaCl and 10% glycerol) were lysed by French Pressure cell (1,500 psi). Cell debris was removed by centrifugation at 20,000 × *g* for 1 h twice. The supernatant was passed through a Ni-Sepharose FF column (Cytiva), pre-equilibrated with 20 mM Im in buffer A. The column was washed with 40 mM Im in buffer A and the His_6_-tagged PlyCYU was eluted with 400 mM Im in buffer A. The His_6_-tagged PlyCYU fractions were concentrated and buffer-exchanged to 50 mM HEPES buffer pH 7.5 containing 50 mM NaCl, 20% glycerol, and 10 mM DTT using a 100 kDa MWCO Amicon ultracentrifugal unit. Other PlyCYU variants—PlyCYU214 (26.0 kDa), PlyCYU277 (32.6 kDa), cyuLyz2 (24.9 kDa), CW7_Lyz2 (37.9 kDa), PlyCYU-Cys34Ala/Ser, and PlyCYU-His99Ala—were purified in a similar manner as His-tagged PlyCYU.

For tag-free PlyCYU (51.8 kDa), cells were suspended in buffer T (100 mM Tris buffer pH 8.0 and 10% vol/vol glycerol) and lysed by French Pressure cell at 1,500 psi. After centrifugation at 20,000 × *g* for 1 h, the crude extract was loaded onto buffer T pre-equilibrated Q-Sepharose column (Cytiva). The column was washed with buffer T. The tag-free PlyCYU did not bind to Q-Sepharose, and the fractions containing PlyCYU with greater than 85% purity were pooled and concentrated.

The purity of all purified proteins studied was assessed by SDS-PAGE, and the protein concentration was determined by Bradford reagent using bovine serum albumin (BSA) as a standard protein.

### Effect of pH and metal ion on PlyCYU activity

The influence of pH and divalent metal ions on PlyCYU activity was investigated using turbidity reduction assay. Briefly, *S. agalactiae* cultured to an early log phase (~2.5 h) at OD_600_ of 0.6 in poly-buffer (50 mM MES, 25 mM sodium acetate, and 50 mM Tris-Cl) at various pH was used as the cell substrate. For testing the effect of pH, a pH range of 6.0–9.5 was used. For testing the effect of divalent metal ions, 0–10 mM of MgCl_2_ or CaCl_2_ was used. The reaction was started by the addition of endolysin (0.3 µM for testing pH effect and 2 µM for testing divalent metal ion effect). OD_600_ was monitored at 37°C for 15 min. The catalytic activity was calculated from ΔOD_600_ per min based on a steady-state kinetic plot. Untreated cell substrate was included as a negative control. The experiments were performed in triplicate. The data were presented as plots of average % relative activity with error bars representing standard deviation against pH values or concentration of MgCl_2_ or CaCl_2_.

### Thermofluor stability assay

To determine the stability of PlyCYU, T_m_ of the protein in various buffer conditions was determined by thermofluor stability assay using SYPRO orange (40). In brief, 20 µL of the enzyme for the final concentration of 5 µM PlyCYU in various buffer conditions (pH 5–9, NaCl 50 mM–300 mM, and 20% glycerol) was mixed with 2 µL 24 × SYPRO orange dye (Invitrogen). The mixtures were subjected to thermal denaturation from 20–100°C at 0.5°C per 30 second increment using a real-time PCR thermocycler (CFX96, Bio-Rad). FRET fluorescence signals were monitored. T_m_ was determined from a derivative plot of temperature and fluorescence.

### Viable cell count assay

*In vitro* antibacterial activities of endolysins were determined by viable cell count assay using broth microdilution method. Fifty microliters of 1 × 10^6^ cfu/mL bacteria suspension in CAMHB (Difco Laboratories) supplemented with 25% (vol/vol) horse serum (Gibco) were added to 50 µL of endolysin. A twofold dilution series of endolysin concentration was tested. The cells were incubated at 37°C for 6 h, serially diluted, and spotted onto BHI agar plates. After 24 h incubation at 37°C, the number of colonies was determined. Antibacterial activity was reported as MBC, the minimum concentration of an antibacterial agent that reduces the viability of initial bacterial inoculum by ≥99.9% or three logarithms. MBC values were reported in µM units to address the disparity in size between endolysins and antibiotics. The values reported are representative of results obtained from at least three independent experiments.

For antibacterial activity in milk, UHT-sterilized whole-fat milk was used as a medium for *S. agalactiae* serotype II suspension, for comparison with CAMHB supplemented with 25% vol/vol horse serum. Ninety microliters of 5.5 × 10^5^ cfu/mL of *S. agalactiae* serotype II suspension in UHT milk were added to 10 µL PlyCYU (25-250 µM), and the mixture was incubated at 37°C for 1, 3, and 6 h. Then, samples were serially diluted and spotted onto BHI agar. The plate was incubated at 37°C for 24 h, and viable cells were counted. The MBC values reported are the values from triplicate experiments. PBS (phosphate buffered saline pH 7.4; 137 mM NaCl, 2.7 mM KCl, 8 mM Na_2_HPO_4_, and 2 mM KH_2_PO_4_) was used instead of PlyCYU solution in the non-treated control group. The data were analyzed using two-tailed unpaired *t*-test in GraphPad Prism 7.0 (GraphPad software), with a *P*-value ≤0.05 considered statistically significant.

### Time-kill kinetics assay

*S. agalactiae* (serotypes Ia, II, and III) resuspended in CAMHB supplemented with 25% (vol/vol) horse serum at 1 × 10^6^ cfu/mL (50 µL) was treated with 50 µL of PlyCYU or ampicillin. The final concentrations of PlyCYU or ampicillin in the wells ranged from 1 × to 4 × MBC of these substances. The MBC of ampicillin against three serotypes is similar, which is 0.31 µM. The reactions were incubated at 37°C and viable cell counts were carried out every hour by microdilution assay. The values reported are the geometric mean of triplicate experiments.

### SEM

The morphology of *S. agalactiae* treated with PlyCYU endolysin was observed by SEM. Briefly, *S. agalactiae* at 10^5^ cfu/mL were incubated with 0.5 or 2.5 µM PlyCYU for 5 or 10 min at 25°C. Treated cells (5 µL) were spotted onto a poly-L-lysine-coated mica sheet and air-dried overnight. The samples were subsequently mounted onto stubs, sputter coated with Au, and visualized by SEM (Schottky FE-SEM SU5000 at 3.0 kV). Untreated bacteria were also included as controls for comparison.

### Park Johnson assay

To evaluate the glucosaminidase activity of the Lyz2 domain, the released reducing sugars were quantified by Park Johnson assay with slight modification (47). A 180 µL of the early log phase of *S. agalactiae* serotype II in PBS at an OD_600_ ~0.6 was added with 20 µL of various PlyCYU variants. The reaction was incubated at 37°C for 30 min. Following incubation, the reaction was centrifuged (15,000 × *g*) and filtered through 10 kDa MWCO Amicon ultracentrifugal unit. A 125 µL aliquot of filtrate was mixed with 125 µL carbonate-cyanide solution (50 mM Na_2_CO_3_ and 10 mM KCN) and 125 µL ferricyanide solution {1 mM K_3_[Fe(CN)_6_]}. The reaction was boiled for 15 min, cooled down, and added with 625 µL ferric iron solution [3 mM (NH_4_)Fe(SO_4_)_2_⋅12H_2_O, 0.1% (wt/vol) SDS, and 0.05 N H_2_SO_4_]. The reaction was incubated at room temperature for 15 min, then the absorbance at 690 nm (A_690_) was read. The amount of reducing sugar released was calculated based on the glucose calibration curve. The lysis reactions of *M. lysodeikticus* (ATCC no. 4698; Sigma) by chicken egg white lysozyme were included as a positive control. All experiments were performed in triplicate.

### SEC-MALS and SEC-UV

SEC-MALS experiments were performed at TPS13A (57, 58), National Synchrotron Radiation Research Center, Republic of China (Taiwan). The SEC analytical column with an Agilent 1260 Infinity II HPLC system (Agilent Technologies) and diode array detector was coupled to an in-line DAWN HELEOS-II MALS and Optilab differential refractive index detectors (Wyatt Technology Corporation). The column and the buffer mobile phase were Superdex 200 Increase 5/150 Gl (Cytiva) and 20 mM sodium phosphate, pH 7.0, with 150 mM NaCl. The injection volume for each protein was 10 µL at 10 mg/mL, at 0.2 mL/min flow rate. Data analysis was performed using the ASTRA software (Wyatt Technology).

In addition, the molecular masses of PlyCYU variants (0.1 mg/mL–0.4 mg/mL) were determined with Superdex 200 Increase 10/300 Gl (GE Healthcare) column equipped with AKTA FPLC system (GE Healthcare) using 50 mM phosphate buffer, pH 7.5, and 150 mM NaCl as a mobile phase at 0.5 mL/min flow rate. A calibration curve of the relative volume ratios (V_e_/V_o_) versus the logarithms of the known molecular weights of standard proteins—thyroglobulin (669 kDa), ferritin (440 kDa), aldolase (158 kDa), conalbumin (75 kDa), ovalbumin (43 kDa), carbonic anhydrase (29 kDa), ribonuclease (13.7 kDa), and aprotinin (6.5 kDa)—was plotted using blue dextran (2,000 kDa) for void volume (V_o_). Protein elution was followed by an absorbance at 280 nm (A_280_) and an elution volume (V_e_) was measured. The molecular masses of PlyCYU variants were calculated from the calibration curve.

## SUPPLEMENTAL MATERIAL

Supplemental material aem.01872-24-s0001.pdf Tables S1 to S3; Fig. S1 to S4.

ASM does not own the copyrights to Supplemental Material that may be linked to, or accessed through, an article. The authors have granted ASM a non-exclusive, world-wide license to publish the Supplemental Material files. Please contact the corresponding author directly for reuse.

## References

[1] Mhalu FS. 1976. Infection with Streptococcus agalactiae in a London hospital. J Clin Pathol 29:309–312. doi:10.1136/jcp.29.4.309 777044 PMC476053

[2] Mian GF, Godoy DT, Leal CAG, Yuhara TY, Costa GM, Figueiredo HCP. 2009. Aspects of the natural history and virulence of S. agalactiae infection in Nile tilapia. Vet Microbiol 136:180–183. doi:10.1016/j.vetmic.2008.10.016 19042097

[3] Pereira UP, Mian GF, Oliveira ICM, Benchetrit LC, Costa GM, Figueiredo HCP. 2010. Genotyping of Streptococcus agalactiae strains isolated from fish, human and cattle and their virulence potential in Nile tilapia. Vet Microbiol 140:186–192. doi:10.1016/j.vetmic.2009.07.025 19726142

[4] Porter JJ, Campbell HM. 1946. Dosages of penicillin for Streptococcus agalactiae mastitis. J Am Vet Med Assoc 109:60–64. 20988133

[5] Liu Y, Liu J. 2022. Group B Streptococcus: virulence factors and pathogenic mechanism. Microorganisms 10:2483. doi:10.3390/microorganisms10122483 36557736 PMC9784991

[6] Balasubramanian N, Pounpandi P, Varatharaju G, Shanmugaiah V, Balakrishnan K, Thirunarayan MA. 2023. Distribution of virulence genes and biofilm characterization of human isolates of Streptococcus agalactiae: a pilot study. Colloids Surf B Biointerfaces 223:113151. doi:10.1016/j.colsurfb.2023.113151 36738701

[7] Zastempowska E, Twarużek M, Grajewski J, Lassa H. 2022. Virulence factor genes and cytotoxicity of Streptococcus agalactiae isolated from bovine mastitis in Poland. Microbiol Spectr 10:e0222421. doi:10.1128/spectrum.02224-21 35608349 PMC9241884

[8] Polianciuc SI, Gurzău AE, Kiss B, Ştefan MG, Loghin F. 2020. Antibiotics in the environment: causes and consequences. Med Pharm Rep 93:231–240. doi:10.15386/mpr-1742 32832887 PMC7418837

[9] Dilrukshi N, Kottahachchi J, Dissanayake T, Fernando N. 2023. Antibiotic sensitivity of Group B Streptococcus from pregnant mothers and its association with resistance genes. Med Princ Pract 32:126–132. doi:10.1159/000530525 37023724 PMC10319089

[10] Han G, Zhang B, Luo Z, Lu B, Luo Z, Zhang J, Wang Y, Luo Y, Yang Z, Shen L, Yu S, Cao S, Yao X. 2022. Molecular typing and prevalence of antibiotic resistance and virulence genes in Streptococcus agalactiae isolated from Chinese dairy cows with clinical mastitis. PLoS One 17:e0268262. doi:10.1371/journal.pone.0268262 35522690 PMC9075616

[11] He Y, Huang J-L, Wang K-Y, Chen D-F, Geng Y, Huang X-L, Ou-Yang P, Zhou Y, Wang J, Min J, Lai W-M. 2017. Pathogenicity of Streptococcus agalactiae in Oreochromis niloticus. Oncotarget 5:s401–s413. doi:10.18632/oncotarget.23551

[12] Li C, Sapugahawatte DN, Yang Y, Wong KT, Lo NWS, Ip M. 2020. Multidrug-resistant Streptococcus agalactiae strains found in human and fish with high penicillin and cefotaxime non-susceptibilities. Microorganisms 8:1055. doi:10.3390/microorganisms8071055 32708529 PMC7409034

[13] Simoni S, Vincenzi C, Brenciani A, Morroni G, Bagnarelli P, Giovanetti E, Varaldo PE, Mingoia M. 2018. Molecular characterization of italian isolates of fluoroquinolone-resistant Streptococcus agalactiae and relationships with chloramphenicol resistance. Microb Drug Resist 24:225–231. doi:10.1089/mdr.2017.0139 28783417

[14] Safari D, Gultom SM, Tafroji W, Azzahidah A, Soesanti F, Khoeri MM, Prayitno A, Pimenta FC, Gloria Carvalho M, Uiterwaal C, Putri ND. 2021. Prevalence, serotype and antibiotic susceptibility of Group B Streptococcus. PLoS One 16:e0252328. doi:10.1371/journal.pone.0252328 34043711 PMC8158947

[15] Murray E, Draper LA, Ross RP, Hill C. 2021. The advantages and challenges of using endolysins in a clinical setting. Viruses 13:680. doi:10.3390/v13040680 33920965 PMC8071259

[16] Vander Elst N. 2024. Bacteriophage-derived endolysins as innovative antimicrobials against bovine mastitis-causing streptococci and staphylococci: a state-of-the-art review. Acta Vet Scand 66:20. doi:10.1186/s13028-024-00740-2 38769566 PMC11106882

[17] Loeffler JM, Nelson D, Fischetti VA. 2001. Rapid killing of Streptococcus pneumoniae with a bacteriophage cell wall hydrolase. Science 294:2170–2172. doi:10.1126/science.1066869 11739958

[18] Fischetti VA. 2008. Bacteriophage lysins as effective antibacterials. Curr Opin Microbiol 11:393–400. doi:10.1016/j.mib.2008.09.012 18824123 PMC2597892

[19] Schmelcher M, Donovan DM, Loessner MJ. 2012. Bacteriophage endolysins as novel antimicrobials. Future Microbiol 7:1147–1171. doi:10.2217/fmb.12.97 23030422 PMC3563964

[20] Cheng Q, Fischetti VA. 2007. Mutagenesis of a bacteriophage lytic enzyme PlyGBS significantly increases its antibacterial activity against group B streptococci. Appl Microbiol Biotechnol 74:1284–1291. doi:10.1007/s00253-006-0771-1 17186236

[21] Donovan DM, Foster-Frey J, Dong S, Rousseau GM, Moineau S, Pritchard DG. 2006. The cell lysis activity of the Streptococcus agalactiae bacteriophage B30 endolysin relies on the cysteine, histidine-dependent amidohydrolase/peptidase domain . Appl Environ Microbiol 72:5108–5112. doi:10.1128/AEM.03065-05 16820517 PMC1489305

[22] Donovan DM, Foster-Frey J. 2008. LambdaSa2 prophage endolysin requires Cpl-7-binding domains and amidase-5 domain for antimicrobial lysis of streptococci. FEMS Microbiol Lett 287:22–33. doi:10.1111/j.1574-6968.2008.01287.x 18673393

[23] Pritchard D.G, Dong S, Kirk MC, Cartee RT, Baker JR. 2007. LambdaSa1 and LambdaSa2 prophage lysins of Streptococcus agalactiae . Appl Environ Microbiol 73:7150–7154. doi:10.1128/AEM.01783-07 17905888 PMC2168211

[24] Pritchard David G., Dong S, Baker JR, Engler JA. 2004. The bifunctional peptidoglycan lysin of Streptococcus agalactiae bacteriophage B30. Microbiology (Reading, Engl) 150:2079–2087. doi:10.1099/mic.0.27063-0 15256551

[25] Fischetti VA, Schmitz J, Gilmer D, Euler C. 2021. Streptococcus bacteriophage lysins for detection and treatment of Gram positive bacteria. US patent 11155799, issued

[26] Oechslin F, Daraspe J, Giddey M, Moreillon P, Resch G. 2013. In vitro characterization of PlySK1249, a novel phage lysin, and assessment of its antibacterial activity in a mouse model of Streptococcus agalactiae bacteremia. Antimicrob Agents Chemother 57:6276–6283. doi:10.1128/AAC.01701-13 24100496 PMC3837886

[27] Gilmer DB, Schmitz JE, Euler CW, Fischetti VA. 2013. Novel bacteriophage lysin with broad lytic activity protects against mixed infection by Streptococcus pyogenes and methicillin-resistant Staphylococcus aureus. Antimicrob Agents Chemother 57:2743–2750. doi:10.1128/AAC.02526-12 23571534 PMC3716137

[28] Vander Elst N, Linden SB, Lavigne R, Meyer E, Briers Y, Nelson DC. 2020. Characterization of the bacteriophage-derived endolysins PlySs2 and PlySs9 with in vitro lytic activity against bovine mastitis Streptococcus uberis. Antibiotics (Basel) 9:621. doi:10.3390/antibiotics9090621 32961696 PMC7558826

[29] Liu G, Zhang S, Gao T, Mao Z, Shen Y, Pan Z, Guo C, Yu Y, Yao H. 2022. Identification of a novel broad-spectrum endolysin, Ply0643, with high antibacterial activity in mouse models of streptococcal bacteriaemia and mastitis. Res Vet Sci 143:41–49. doi:10.1016/j.rvsc.2021.12.014 34973538

[30] Bocanova L, Psenko M, Barák I, Halgasova N, Drahovska H, Bukovska G. 2022. A novel phage-encoded endolysin EN534-C active against clinical strain Streptococcus agalactiae GBS. J Biotechnol 359:48–58. doi:10.1016/j.jbiotec.2022.09.016 36179792

[31] Vander Elst N, Bert J, Favoreel H, Lavigne R, Meyer E, Briers Y. 2023. Development of engineered endolysins with in vitro intracellular activity against streptococcal bovine mastitis-causing pathogens. Microb Biotechnol 16:2367–2386. doi:10.1111/1751-7915.14339 37853918 PMC10686134

[32] Indiani C, Sauve K, Raz A, Abdelhady W, Xiong YQ, Cassino C, Bayer AS, Schuch R. 2019. The antistaphylococcal Lysin, CF-301, activates key host factors in human blood to potentiate methicillin-resistant Staphylococcus aureus bacteriolysis. Antimicrob Agents Chemother 63:e02291-18. doi:10.1128/AAC.02291-18 30670427 PMC6437495

[33] Oh JT, Cassino C, Schuch R. 2019. Postantibiotic and sub-MIC effects of exebacase (Lysin CF-301) enhance antimicrobial activity against Staphylococcus aureus. Antimicrob Agents Chemother 63:e02616-18. doi:10.1128/AAC.02616-18 30936103 PMC6535547

[34] Eichenseher F, Herpers BL, Badoux P, Leyva-Castillo JM, Geha RS, Zwart M, McKellar J, Janssen F, Rooij B, Selvakumar L, Röhrig C, Frieling J, Offerhaus M, Loessner MJ, Schmelcher M. 2022. Linker-improved chimeric endolysin selectively kills Staphylococcus aureus in vitro. Antimicrob Agents Chemother:e0227321. doi:10.1128/aac.02273-21 35416713 PMC9112974

[35] Cheng Q, Nelson D, Zhu S, Fischetti VA. 2005. Removal of group B streptococci colonizing the vagina and oropharynx of mice with a bacteriophage lytic enzyme. Antimicrob Agents Chemother 49:111–117. doi:10.1128/AAC.49.1.111-117.2005 15616283 PMC538902

[36] Modh RH, Islam MM, Nauriyal DS, Modi RJ, Wadhwani KN. 2018. Study on pH and somatic cell count in milk of sub-clinical mastitic cows in association with udder and teat shape. Indian J Anim Prod Mgmt 34:75–79.

[37] Oh HE, Deeth HC. 2017. Magnesium in milk. Int Dairy J 71:89–97. doi:10.1016/j.idairyj.2017.03.009

[38] Gaucheron F. 2005. The minerals of milk. Reprod Nutr Dev 45:473–483. doi:10.1051/rnd:2005030 16045895

[39] Holt C, Dalgleish DG, Jenness R. 1981. Calculation of the ion equilibria in milk diffusate and comparison with experiment. Anal Biochem 113:154–163. doi:10.1016/0003-2697(81)90059-2 7270880

[40] Lavinder JJ, Hari SB, Sullivan BJ, Magliery TJ. 2009. High-throughput thermal scanning: a general, rapid dye-binding thermal shift screen for protein engineering. J Am Chem Soc 131:3794–3795. doi:10.1021/ja8049063 19292479 PMC2701314

[41] Yan J, Yang R, Yu S, Zhao W. 2021. The application of the lytic domain of endolysin from Staphylococcus aureus bacteriophage in milk. J Dairy Sci 104:2641–2653. doi:10.3168/jds.2020-19456 33358804

[42] Schmelcher M, Powell AM, Camp MJ, Pohl CS, Donovan DM. 2015. Synergistic streptococcal phage λSA2 and B30 endolysins kill streptococci in cow milk and in a mouse model of mastitis. Appl Microbiol Biotechnol 99:8475–8486. doi:10.1007/s00253-015-6579-0 25895090 PMC4573782

[43] Verbree CT, Dätwyler SM, Meile S, Eichenseher F, Donovan DM, Loessner MJ, Schmelcher M. 2018. Corrected and republished from: identification of peptidoglycan hydrolase constructs with synergistic staphylolytic activity in cow’s milk. Appl Environ Microbiol 84:e02134-17. doi:10.1128/AEM.02134-17 PMC5734024 29320762

[44] Jumper J, Evans R, Pritzel A, Green T, Figurnov M, Ronneberger O, Tunyasuvunakool K, Bates R, Žídek A, Potapenko A, et al.. 2021. Highly accurate protein structure prediction with AlphaFold. Nature 596:583–589. doi:10.1038/s41586-021-03819-2 34265844 PMC8371605

[45] Varadi M, Anyango S, Deshpande M, Nair S, Natassia C, Yordanova G, Yuan D, Stroe O, Wood G, Laydon A, et al.. 2022. AlphaFold protein structure database: massively expanding the structural coverage of protein-sequence space with high-accuracy models. Nucleic Acids Res 50:D439–D444. doi:10.1093/nar/gkab1061 34791371 PMC8728224

[46] Holm L. 2020. DALI and the persistence of protein shape. Protein Sci 29:128–140. doi:10.1002/pro.3749 31606894 PMC6933842

[47] Park JT, Johnson MJ. 1949. A submicrodetermination of glucose. Journal of Biological Chemistry 181:149–151. doi:10.1016/S0021-9258(18)56635-7 15390401

[48] D’Atri V, Imiołek M, Quinn C, Finny A, Lauber M, Fekete S, Guillarme D. 2024. Size exclusion chromatography of biopharmaceutical products: From current practices for proteins to emerging trends for viral vectors, nucleic acids and lipid nanoparticles. J Chromatogr A 1722:464862. doi:10.1016/j.chroma.2024.464862 38581978

[49] Son B, Kong M, Ryu S. 2018. The Auxiliary role of the amidase domain in cell wall binding and exolytic activity of staphylococcal phage endolysins. Viruses 10:284. doi:10.3390/v10060284 29799482 PMC6024855

[50] Oechslin F, Menzi C, Moreillon P, Resch G. 2021. The multidomain architecture of a bacteriophage endolysin enables intramolecular synergism and regulation of bacterial lysis. J Biol Chem 296:100639. doi:10.1016/j.jbc.2021.100639 33838182 PMC8144678

[51] McGowan S, Buckle AM, Mitchell MS, Hoopes JT, Gallagher DT, Heselpoth RD, Shen Y, Reboul CF, Law RHP, Fischetti VA, Whisstock JC, Nelson DC. 2012. X-ray crystal structure of the streptococcal specific phage lysin PlyC. Proc Natl Acad Sci USA 109:12752–12757. doi:10.1073/pnas.1208424109 22807482 PMC3412044

[52] Proença D, Velours C, Leandro C, Garcia M, Pimentel M, São-José C. 2015. A two-component, multimeric endolysin encoded by a single gene. Mol Microbiol 95:739–753. doi:10.1111/mmi.12857 25388025

[53] Lu S, Wang J, Chitsaz F, Derbyshire MK, Geer RC, Gonzales NR, Gwadz M, Hurwitz DI, Marchler GH, Song JS, Thanki N, Yamashita RA, Yang M, Zhang D, Zheng C, Lanczycki CJ, Marchler-Bauer A. 2020. CDD/SPARCLE: the conserved domain. Nucleic Acids Res 48:D265–D268. doi:10.1093/nar/gkz991 31777944 PMC6943070

[54] Saitou N, Nei M. 1987. The neighbor-joining method: a new method for reconstructing phylogenetic trees. Mol Biol Evol 4:406–425. doi:10.1093/oxfordjournals.molbev.a040454 3447015

[55] Tamura K, Stecher G, Kumar S. 2021. MEGA11: Molecular evolutionary genetics analysis version 11. Mol Biol Evol 38:3022–3027. doi:10.1093/molbev/msab120 33892491 PMC8233496

[56] Studier FW. 2005. Protein production by auto-induction in high density shaking cultures. Protein Expr Purif 41:207–234. doi:10.1016/j.pep.2005.01.016 15915565

[57] Liu D-G, Chang C-H, Chiang L-C, Lee M-H, Chang C-F, Lin C-Y, Liang C-C, Lee T-H, Lin S-W, Liu C-Y, et al.. 2021. Optical design and performance of the biological small-angle X-ray scattering beamline at the Taiwan photon source. J Synchrotron Radiat 28:1954–1965. doi:10.1107/S1600577521009565 34738951 PMC8570220

[58] Shih O, Liao K-F, Yeh Y-Q, Su C-J, Wang C-A, Chang J-W, Wu W-R, Liang C-C, Lin C-Y, Lee T-H, Chang C-H, Chiang L-C, Chang C-F, Liu D-G, Lee M-H, Liu C-Y, Hsu T-W, Mansel B, Ho M-C, Shu C-Y, Lee F, Yen E, Lin T-C, Jeng U. 2022. Performance of the new biological small- and wide-angle X-ray scattering beamline 13A at the Taiwan Photon Source. J Appl Crystallogr 55:340–352. doi:10.1107/S1600576722001923 35497659 PMC8985603

