# Supplementary material for: PlyCYU endolysin targeting Streptococcus agalactiae exhibits a CHAP activity and a glucosaminidase domain mediating multimerization
Source: Appl Environ Microbiol. 2025 Aug 19;91(9):e01872-24. doi: 10.1128/aem.01872-24 (PMC12442389; doi:10.1128/aem.01872-24)
Supplement: Supplemental material — Tables S1 to S3; Fig. S1 to S4. [file aem.01872-24-s0001.pdf]

Supplementary materials for

PlyCYU endolysin targeting *Streptococcus agalactiae* exhibits a CHAP activity and a glucosaminidase domain mediating multimerization

Sakunrat Ubonprasert,[1] Wachiraporn Wachiradusit,[1] Wichai Pornthanakasem,[1] Warangkhana Songsungthong,[1] Aritsara Jaruwat,[1] Sasina Premjaichon,[2] Tanaporn Uengwetwanit,[1] Rinrada Suntivich,[1] Konrawee Thananon,[3] Kanyarat Suksomjaisaman,[3] Jeerus Sucharitakul,[3] Chutathip Puyprom,[4,5] Tamonwan Lotangchanintra,[4,5] Kanokwan Salamteh,[4,5] Kittikhun Wangkanont,[4,5] Channarong Rodkhum,[6] Wonnop Visessanguan,[7,8] Pimchai Chaiyen,[2] Penchit Chitnumsub,[1,2] Ubolsree Leartsakulpanich[1]

Table S1 Antibacterial activities by viable cell count assay at various concentrations of (a) PlyCYU, (b) PlyCYU-Cys34Ser and (c) PlyCYU-Cys34Ala and PlyCYU-His99Ala

(a)

PlyCYU (μM)	Average cell number (cfu/mL)		
	Sag Ia	Sag II	Sag III
0	2.73E+08	2.31E+08	1.91E+08
0.313	ND	2.10E+06	ND
0.625	ND	9.67E+05	ND
1.25	ND	1.20E+05	ND
2.5	6.00E+07	6.67E+03	ND
5	1.50E+06	1.00E+02	ND
10	8.67E+04	ND	2.03E+06
20	4.00E+03	ND	9.67E+05
40	1.00E+02	ND	1.10E+05
80	ND	ND	3.00E+04
100	ND	ND	ND

ND not determined

(b)

PlyCYU-Cys34Ser (μM)	Average cell number (cfu/mL)		
	Sag Ia	Sag II	Sag III
0	2.73E+08	2.31E+08	1.91E+08
20	2.53E+08	2.20E+08	1.80E+08
80	2.60E+08	2.13E+08	1.73E+08
100	2.57E+08	2.10E+08	1.70E+08

(c)

[Endolysin] (μM)	Average Sag II number (cfu/mL) upon treated with	
	PlyCYU-Cys34Ala	PlyCYU-His99Ala
0	2.19E+08	2.19E+08
1.25	1.87E+08	2.03E+08
2.5	2.17E+08	1.93E+08
5	2.07E+08	2.00E+08
50	1.97E+08	1.97E+08
100	2.03E+08	2.07E+08

Table S2 Quantitation of reducing sugar released from the lysis reactions of *S. agalactiae* serotype II with PlyCYU variants and *M. lysodeikticus* with lysozyme using Park Johnson assay.

Endolysin	Reducing sugar (μM)
PlyCYU (10 μM)	10.3 ± 0.3
PlyCYU-Cys34Ala (20 and 50 μM)	0
PlyCYU-His99Ala (50 μM)	0
PlyCYU277 (20 μM)	0
cyuLyz2 (20 and 80 μM)	0
CW7_Lyz2 (20 and 80 μM)	0
PlyCYU277 (20 μM) + cyuLyz2 (20 μM)	3.1 ± 0.4
PlyCYU277 (20 μM) + CW7_Lyz2 (20 μM)	0
Lysozyme (0.35 μM)	9.2 ± 0.5

Table S3 Primers used in this study.

PlyCYU variants	Amino acid sequence number	Primers
PlyCYU277	1-277	CYU-NdeI_F (5’-CCCCCATATGACCATCAACCTGGAAACCA-3’)<br>CYU277-XhoI R (5’-CCCCCTCGAGTTACAGATCACCTTCCTCTTTGTTCA-3’)
PlyCYU214	1-214	CYU-NdeI_F (5’-CCCCCATATGACCATCAACCTGGAAACCA-3’)<br>CYU214-BamHI_R (5’-GATACAGGATCCTTAGCTTTTCTTACGCTCCTCGCC-3’)
PlyCYU150	1-150	CYU-NdeI_F (5’-CCCCCATATGACCATCAACCTGGAAACCA-3’)<br>CYU150-XhoI_R (5’-GGGGCTCGAGTTACTCCAGGCTCTTGTTGCTGATG-3’)
cyuLyz2	266-473	Lyz2-NdeI_F (5’-GGGGCATATGGGTAACGCGACCGTGAACAA-3’)<br>Lyz2-XhoI_R (5’-GGGGCTCGAGTTAAACCGGTTTCTTTTCCAGACG-3’)
CW7_Lyz2	142-473	NdeI_CYU_P142_F (5’-GGGGCATATGCCGAGCATCAGCAACAAGAG-3’)<br>CYUstop-BamHI _R (5’-GGGGGGATCCTTAAACCGGTTTCTTTTCCAGACG-3’)
PlyCYU-Cys34Ala	1-473, Cys34Ala	34A-F (5’-CCGTAACGGCCCGAACAGCTACGATGCCAGCAGCGCGGTGTACTATGCGCT-3’)<br>34A-R (5’-AGCGCATAGTACACCGCGCTGCTGGCATCGTAGCTGTTCGGGCCGTTACGG-3’)
PlyCYU-Cys34Ser	1-473, Cys34Ser	34S-F (5’-CTACCGTAACGGCCCGAACAGCTACGATAGCAGCAGCGCGGTGTACTATGCGCT-3’)<br>34S-R (5’-AGCGCATAGTACACCGCGCTGCTGCTATCGTAGCTGTTCGGGCCGTTACGGTAG-3’)
PlyCYU-His99Ala	1-473, His99Ala	99F (5’-GCTACAGCAGCGGTGAAGGTGGCGCCACCGGCATTTTCGTGGACAACGTTAACAT-3’)<br>99R (5’-ATGTTAACGTTGTCCACGAAAATGCCGGTGGCGCCACCTTCACCGCTGCTGTAGC-3’)

Fig. S1 Endolysins containing amidase_5 and cpl-7-glucosaminidase identified from *S. suis* genomes in this study–CYU89965.1 (473 amino acids), CYV20156.1 (473 amino acids), CYW17827.1 (465 amino acids) , YP950557.1 (481 amino acids), AGZ23278.1 (451 amino acids), WP023370479.1 (451 amino acids), CYW19138.1 (465 amino acids), and AGF87701.1 (452 amino acids). Identical amino acid sequences but different accession numbers were noted for WP023370479.1 with AGZ23278.1, and CYU89965.1 with CYV20156.1. (a) Schematic showing modular structure of these endolysins, which contain N-terminal amidase_5 domain, followed by two cpl-7 cell wall binding motifs belonging to the CW_7 superfamily, then C-terminal glucosaminidase Lyz2 domain. (b) Multiple amino acid sequence alignments showing different lengths of linker tethering the second cpl-7 motif and the glucosaminidase domain. The linker regions are boxed. Numbers in brackets indicate percent identity to PlyCYU. (*) indicates identical residue, (:) indicates conserved substitution, (.) indicates semi-conserved substitution, and unmarked positions indicate non-conserved substitution.

(a)

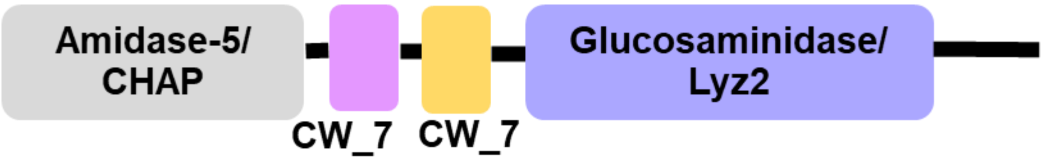

(b)

```
CYU89965.1                MTINLETSIRWMSDRVGKVSYSMDYRNGPNSYDCSSAVYYALMAGGAISAGWAVNTEYMH
CYV20156.1 [100]          MTINLETSIRWMSDRVGKVSYSMDYRNGPNSYDCSSAVYYALMAGGAISAGWAVNTEYMH
CYW17827.1 [88]           MTINLETSIRWMSDRVGKVSYSMDYRNGPNSYDCSSAVYYALMAGGAISAGWAVNTEYMH
YP950557.1 [78]           MTINIETAIRWMTDRVGLVKYSMDYRNGPNSFDCSSSVYYALMAGGAISAGWAVNTEYEH
AGZ23278.1 [80]           MTINLETSIRWMSDRVGKVSYSMDYRNGPNSYDCSSAVYYALMAGGAISAGWAVNTEHMH
WP023370479.1 [80]        MTINLETSIRWMSDRVGKVSYSMDYRNGPNSYDCSSAVYYALMAGGAISAGWAVNTEHMH
CYW19138.1 [68]           MGVNIETALRWMSDRKGRVTYSMDYRNGPNSFDCSSSVYYALMSAGAISAGWAVNTEYEH
AGF87701   [77]           MTINLETSIRWMSDRVGKVSYSMDYRNGPNSYDCSSAVYYALMAGGAISAGWAVNTEYMH
                          * :*:**::***:** * *.***********:****:******:.************: *

CYU89965.1                DWLIRNGYVLVAENKPFNAKRHDVFIWGKRGYSSGEGGHTGIFVDNVNIIHCNFKRNGIT
CYV20156.1                DWLIRNGYVLVAENKPFNAKRHDVFIWGKRGYSSGEGGHTGIFVDNVNIIHCNFKRNGIT
CYW17827.1                DWLIRNGYVLVAENKPFNAQRHDVFIWGKRGYSSGEGGHTGIFVDNVNIIHCNFKRNGIT
YP950557.1                DWLIKNGYKLIAENQDWDAKRGDIFIWGRRGQSSGAGGHTGIFVDPDNIIHCNYANNSIT
AGZ23278.1                DWLIRNGYVLVAENKPFNAQRHDVCILGKRGYSSGAGGHVVIFVDNVNVIHCNYARNGIT
WP023370479.1             DWLIRNGYVLVAENKPFNAQRHDVCILGKRGYSSGAGGHVVIFVDNVNVIHCNYARNGIT
CYW19138.1                DWLIKNGFTLIAENTDWDAKRGDIFIWGRRGHSAGAGGHTGIFIDPDNIIHCNYARNSIT
AGF87701.1                DWLIRNGYVLVAENKPFNAQRHDVCILGKRGYSSGAGGHVVIFVDNVNVIHCNYARNGIS
                          ****:**: *:***  ::*:* *: * *:** *:* ***. **:*  *:****: .*.*:

CYU89965.1                IDDYNKVSRG---MYYYLYRPANQP---SIS--NKSLEQLVKETLAGVHGNGDARKASLG
CYV20156.1                IDDYNKVSRG---MYYYLYRPANQP---SIS--NKSLEQLVKETLAGVHGNGDARKASLG
CYW17827.1                IDDYNKVSRG---MYYYLYRPVNQP---SIS--NKSLDQLVKETLAGVHGNGDARKASLG
YP950557.1                INNYNQTAAASGWMYCYVYRLGNQP---TTSPAGKTLDTLVKETLAGKYGNGDQRKAALG
AGZ23278.1                IDNYNQVHRG---MYCYLYRPANQPSTS-----NKSLDQLVKETLAGVHGNGDARKASLG
WP023370479.1             IDNYNQVHRG---MYCYLYRPANQPSTS-----NKSLDQLVKETLAGVHGNGDARKASLG
CYW19138.1                VDNYNQTAAASGWMYCYVYRLANQT-----STAGKSLETLVQETLAGKYGNGDTRKAALG
AGF87701.1                IDNYNQVHRG---MYYYLYRPANQP-----SISNKSLDQLVKETLAGVHGNGDTRKASLG
                          :::**:.  .   ** *:**  **.        .*:*: **:***** :**** ***:**

CYU89965.1                NQYEPVMAVINGKATALKKTIDELVQEVIAGKHGNGEERKKSLGSDYDAVQKRVTEILKS
CYV20156.1                NQYEPVMAVINGKATALKKTIDELVQEVIAGKHGNGEERKKSLGSDYDAVQKRVTEILKS
CYW17827.1                NQYEPVMAVINGKATTSQKTVDQLAQEVIAGKHGNGEARKKSLGSQYEAVQKRVTELLK-
YP950557.1                NQYEAVMAVINGKATAPKKTVDQLAQEVIQGKHGNGEDRKKSLGPDYDAVQKRVTEILQG
AGZ23278.1                NQYEPVMAVINSKATASEKTDEELAREVLAGKHGVGEDRKRSLGPRYEPVQAIVNKLLKA
WP023370479.1             NQYEPVMAVINSKATASEKTDEELAREVLAGKHGVGEDRKRSLGPRYEPVQAIVNKLLKA
CYW19138.1                NQYEAVMAVINGKASPSEKSDEELAREVLAGKHGAGEDRKRSLGPRYESVQAKVNELLKA
AGF87701.1                SQYEAVMAVINGKASASEKSDEELAREVLAGKHGAGEDRKRSLGPRYEPVQAKVNELLKA
                          .***.******.**:. :*: ::*.:**: **** ** **:***. *:.**  *.::*:

CYU89965.1                DTSGNTPKTPSDTPKSGAVNSSTEPKIEETEPTGNAT---VNKEEGDLSFNGAILKKAVL
CYV20156.1                DTSGNTPKTPSDTPKSGAVNSSTEPKIEETEPTGNAT---VNKEEGDLSFNGAILKKAVL
CYW17827.1                -------KQPSEPSKAQEVKQPTETKTSQTEPTGNAT---VNKEDGDLSFNGAILKKVVL
YP950557.1                STSGNAPKLASDAPKNEVVNSSTEPKTEETWATGKATDTKITKEDGDLSFNGAILKKSVL
AGZ23278.1                K-------------------------EKPSETVKNEPQTVQFKEDGDLSFNGAILKKSVL
WP_023370479.1            K-------------------------EKPSETVKNEPQTVQFKEDGDLSFNGAILKKSVL
CYW19138.1                K-------------------------EKPSEVVKNEPQTVQFKEDGDLSFNGAILKKSVL
AGF87701.1                K-------------------------EKPSEVVKNEPQTVQFKEDGDLSFNGAILKKSVL
                                                   . :  . : .     **:************ **

CYU89965.1                DKILANCKKHDILPSYALTILHYEGLWGTSAVGKADNNWGGMTWTGQGNRPSGVTVTQGT
CYV20156.1                DKILANCKKHDILPSYALTILHYEGLWGTSAVGKADNNWGGMTWTGQGNRPSGVTVTQGT
CYW17827.1                DKILANCKNHDILPSYALTILHYEGLWGTSAVGKADNNWGGMTWTGQGNRPSGVTVTQGS
YP950557.1                DVILANCKKHDILPSYALTILHYEGLWGTSAVGKADNNWGGMTWTGKGERPSGVTVTQGT
AGZ23278.1                DVILAKCKEHNILPSYAITVLHFEGLWGTSAVGKADNNWGGMTWTGKGERPSGVTVTQGT
WP023370479.1             DVILAKCKEHNILPSYAITVLHFEGLWGTSAVGKADNNWGGMTWTGKGERPSGVTVTQGT
CYW19138.1                EIILKKCKEHDILPSYALTILHYEGLWGTSAVGKADNNWGGMTWTGHGNRPSGVVVTQGL
AGF87701.1                EIILKKCKEHDILPSYALTILHYEGLWGTSAVGKADNNWGGMTWTGQGNRPSGVVVTQGL
                          : ** :**:*:******:*:**:***********************:*:*****.****
```

```
CYU89965.1          DRPSNEGGHYMHYANVDDFLTDWFYLLRVGGSYKVSGAKTFSEAVKGMFKVGGAVYDYAA
CYV20156.1          DRPSNEGGHYMHYANVDDFLTDWFYLLRVGGSYKVSGAKTFSEAVKGMFKVGGAVYDYAA
CYW17827.1          ARPSNEGGHYMHYASVDDFLTDWFYLLRSGGSYKVSGAKTFSEAVKGMFKVGGAVYDYAA
YP_950557.1         ARPACEGGHYMHYASVDDFLTDWFYLLRSGGSYKVSGAKTFSDAVKGMFKIGGAVYDYAA
AGZ23278.1          ARPAYEGGHYMHYASVDDFLTDWFYLLRSGGSYKVSGAKTFSEAVKGMFKVGGAVYDYAA
WP_023370479.1      ARPAYEGGHYMHYASVDDFLTDWFYLLRSGGSYKVSGAKTFSEAVKGMFKVGGAVYDYAA
CYW19138.1          ARPSNEGGHYMHYASVDDFLTDWFYLLRSGGSYKVSGAKTFSESVKGMFQVGGAKYDYAA
AGF87701.1          ARPSNEGGHYMHYASVDDFLTDWFYLLRKDGSYKVSGALTFSESIKGMFQVGGAKYDYAA
                    **: *********.************* .******** ***::****::*** *****

CYU89965.1          SGFDSYIVGASSRLKAIESENGSLDKFDKQTVSDVVQS-DNIEINVEGIEVIINGETYRL
CYV20156.1          SGFDSYIVGASSRLKAIESENGSLDKFDKQTVSDVVQS-DNIEINVEGIEVIINGETYRL
CYW17827.1          SGFDSYIVGASSRLKAIESENGSLAKYDQQTVTDVAKT-DKIEVAIEGIEVTINGTRYKL
YP950557.1          SGFDSYIIGASSRLKAIEAENGSLDKFDKQTVTDVGQS-DKIEVTIEGIEISINGVTYTL
AGZ23278.1          SGFDSYIVGASSRLKAIESENGSLAKYDQQTVTDVAKT-DKIEVAIEGIEVTINGTRYKL
WP023370479.1       SGFDSYIVGASSRLKAIESENGSLAKYDQQTVTDVAKT-DKIEVAIEGIEVTINGTRYKL
CYW19138.1          AGYNSYLVGATSRLKAIESENGSLTRFDATSNNVHSVDPDKISVDIDGIEVTINGVVYKL
AGF87701.1          AGYDSYLVGATSRLKAIESENGSLTRFDATSNNVHSVDPDKISVDIDGIEVTINGVVYKL
                    :*::**::**:*******:***** ::*  : .      *:*.: ::***: ***  * *

CYU89965.1          EKKPV
CYV20156.1          EKKPV
CYW17827.1          TKEPI
YP950557.1          SKKPV
AGZ23278.1          TKEPI
WP023370479.1       TKEPI
CYW19138.1          EKKPV
AGF87701.1          EKKPV
                     *:*:
```

Fig. S2 Comparative analysis of endolysins against *S. agalactiae*. (a) Phylogenetic tree showing the evolutionary relationships among endolysins against *S. agalactiae*–PlyCYU (CYU89965.1), PlySs1 (AGF87701.1), LambdaSa2 (AAN00700.1), LambdaSa1 (AAM99503.1), PlySs2 (CP110141.1), PlySK1249 (EGL49245.1), PlySs9 (AGF87618.1), EN534 (WP017646700.1), and Ply0643 (MK448787.1), B30 (AY149214.3), and PlyGBS (AY428505.1). Evolutionary history was inferred using the Neighbor-Joining method. The tree is drawn and shown with scale bars and bootstrap percentage. Scale bar refers to a phylogenetic distance of 0.2 amino acid substitutions per site. Numbers on the branches indicate bootstrap percentage after 1000 replications in constructing the tree. (b and c) Multiple amino acid sequence alignments of PlyCYU with those endolysins described in (a) that share ≥40% and ~10% sequence identities, respectively. Numbers in brackets indicate percent identity to PlyCYU. (*) indicates identical residue, (:) indicates conserved substitution, (.) indicates semi-conserved substitution, and unmarked positions indicate non-conserved substitution.

(a)

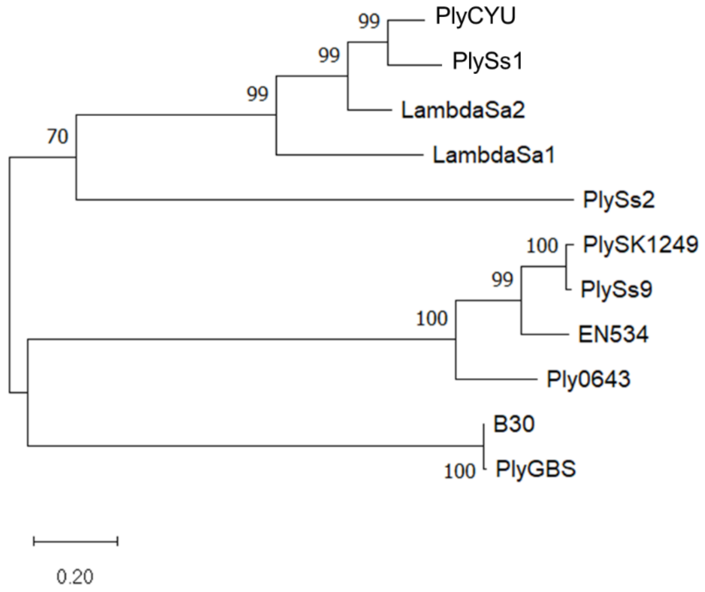

(b)

```
PlyCYU            MTINLETSIRWMSDRVGKVSYSMDYRNGPNSYDCSSAVYYALMAGGAISAGWAVNTEYMH
PlySs1   [77]     MTINLETSIRWMSDRVGKVSYSMDYRNGPNSYDCSSAVYYALMAGGAISAGWAVNTEYMH
LambdaSa2[74]     MEINTEIAIAWMSARQGKVSYSMDYRDGPNSYDCSSSVYYALRSAGASSAGWAVNTEYMH
LambdaSa1[40]     MVINIEQAIAWMASRKGKVTYSMDYRNGPSSYDCSSSVYFALRSAGASDNGWAVNTEYEH
                  * ** * :* **: * ***:******:**.******:**:** :.** . ******** *

PlyCYU            DWLIRNGYVLVAENKPFNAKRHDVFIWGKRGYSSGEGGHTGIFVDNVNIIHCNFKRNGIT
PlySs1            DWLIRNGYVLVAENKPFNAQRHDVCILGKRGYSSGAGGHVVIFVDNVNVIHCNYARNGIS
LambdaSa2         DWLIKNGYELIAENVDWNAVRGDIAIWGMRGHSSGAGGHVVMFIDPENIIHCNWANNGIT
LambdaSa1         DWLIKNGYVLIAENTNWNAQRGDIFIWGKRGASAGAFGHTGMFVDPDNIIHCNYGYNSIT
                  ****:*** *:***  :** * *: * * ** *:*  **. :*:*  *:****:  *.*:

PlyCYU            IDDYNKVSRG---MYYYLYRPANQPSISNKSLEQLVKETLAGVHGNGDARKASLGNQYEP
PlySs1            IDNYNQVHRG---MYYYLYRPANQPSISNKSLDQLVKETLAGVHGNGDTRKASLGSQYEA
LambdaSa2         VNNYNQTAAASGWMYCYVYRLKSGASTQGKSLDTLVKETLAGNYGNGEARKAVLGNQYEA
LambdaSa1         VNNHDEIWGYNGQPYVYAYRYARKQSNAKVDNQSVVSKFEKELDVNTPLSNSNMP-----
                  :::::         * * **     *    . : :*.:        *    :: :

PlyCYU            VMAVINGKATALKKTIDELVQEVIAGKHGNGEERKKSLGSDYDAVQKRVTEILKSDTSGN
PlySs1            VMAVINGKASASEKSDEELAREVLAGKHGAGEDRKRSLGPRYEPVQAKVNELLKAKEK--
LambdaSa2         VMSVINGKTTTNQKTVDQLVQEVIAGKHGNGEARKKSLGSQYDAVQKRVTELLKKQPS--
LambdaSa1         ------------------------------------------------------------

PlyCYU            TPKTPSDTPKSGAVNSSTEPKIEETEPTGNATVNKEEGDLSFNGAILKKAVLDKILANCK
PlySs1            --------------------PSEVVKNEPQTVQFKEDGDLSFNGAILKKSVLEIILKKCK
LambdaSa2         ------EPFKAQEVNKPTETKTSQTELTGQATATKEEGDLSFNGTILKKAVLDKILGNCK
LambdaSa1         ------------------------------------------------------------

PlyCYU            KHDILPSYALTILHYEGLWGTSAVGKADNNWGGMTWTGQGNRPSGVTVTQGTDRPSNEGG
PlySs1            EHDILPSYALTILHYEGLWGTSAVGKADNNWGGMTWTGQGNRPSGVVVTQGLARPSNEGG
LambdaSa2         KHDILPSYALTILHYEGLWGTSAVGKADNNWGGMTWTGQGNRPSGVTVTQGSARPSNEGG
LambdaSa1         ------------------------------------------------------------

PlyCYU            HYMHYANVDDFLTDWFYLLRVGGSYKVSGAKTFSEAVKGMFKVGGAVYDYAASGFDSYIV
PlySs1            HYMHYASVDDFLTDWFYLLRKDGSYKVSGALTFSESIKGMFQVGGAKYDYAAAGYDSYLV
LambdaSa2         HYMHYASVDDFLTDWFYLLRAGGSYKVSGAKTFSEAIKGMFKVGGAVYDYAASGFDSYIV
LambdaSa1         ---------------YYEATISEDYYVESKPDVNSTDKELLVAGTRVRVYEKVKGWARIG
                                 :*    . .* *..   ...: * :: .*     *       : :

PlyCYU            GASSRLKAIESENGSLDKFDKQT-VSDVVQSDNIEINVEGIEVIINGETYRLEKKPV
PlySs1            GATSRLKAIESENGSLTRFDATSNNVHSVDPDKISVDIDGIEVTINGVVYKLEKKPV
LambdaSa2         GASSRLKAIEAENGSLDKFDKATDIGDGSK-DKIDITIEGIEVTINGITYELTKKPV
LambdaSa1         APQSNQWVEDA----------------------------------------------
                  .. *.  . ::
```

(c)

```
PlyCYU              ------------------------------------------------------------
PlySK1249 [11]      ------------------------------------------------------------
PlySs9 [10]         ------------------------------------------------------------
EN534 [12]          ------------------------------------------------------------
Ply0643 [10]        MTFLSKIKDGCLASWEHGILPSVSAAQAILESGWGESLLAQYPNHNLFGIKASSDWKGKR
B30 [11]            ------------------------------------------------------------
PlyGBS [11]         ------------------------------------------------------------
PlySs2 [13]         ------------------------------------------------------------


PlyCYU              ------------------------------------------------------------
PlySK1249           ------------------------------------------------------------
PlySs9              ------------------------------------------------------------
EN534               ------------------------------------------------------------
Ply0643             VDLPTQEYIDGKFVTVEATFRKYDSWEESIKDHALFFSETAWRRSHYQNVLGEEDYKKTC
B30                 ------------------------------------------------------------
PlyGBS              ------------------------------------------------------------
PlySs2              ------------------------------------------------------------


PlyCYU              -------------------------------------------MTINLETSIRWMSDRVG
PlySK1249           ------------------------------------------MGKHLVICGHGQGRTTYD
PlySs9              ------------------------------------------MGKHLVICGHGQGRTGYD
EN534               ------------------------------------------MGKHLVICGHGQGRTTYD
Ply0643             LALQASGYATDPNYGSKLITLIEAHHLNTWDDRILNKKGETTMSKHLVICGHGQGQTGYD
B30                 ----------------------------------------------MATYQEYKSRSNGN
PlyGBS              ----------------------------------------------MATYQEYKSRSNGN
PlySs2              ------------------------------------------MTTVNEALNNVRAQVGSG
                                                                               .

PlyCYU              KVSYSMDYRNGPNSYDCSSAVYYALMAGGAISAGWAVNTEYMHDWLIRNGYVLVAENKPF
PlySK1249           PGAVNAKLGITEAGKVRELAKLMSKYSGQQIDFITEQNVYDYRSITSIGKGYDSITELHF
PlySs9              PGAVNAKLGITEAGKVRELSKLMSKYSGQQIDFITEQNVYDYRSITSIGKGYDSITELHF
EN534               PGAVNSKRGITEAGKVRELARLMSKYSGKNIDYITDQNVYDYKSLAILGKGYDSVTELHF
Ply0643             PGATNPSLGITEAGKVREFANLMKKYSGNRIDYITDHNVYDYRSIGSLGNGYESITELHF
B30                 AYDIDGSFGAQCWDGYADYCKYLGLPYANCTNTGYARDIWEQRHENGILNYFDEVEVMQA
PlyGBS              AYDIDGSFGAQCWDGYADYCKYLGLPYANCTNTGYARDIWEQRHENGILNYFDEVEVMQA
PlySs2              VSVGNGECYALASWYERMISPDATVGLGAGVGWVSGATGDTISAKNIG------------
                        . .            .       .   .

PlyCYU              NAKRHDVFIWGKRGYSSGEGGHTGIFVDNVNIIHCNFKRNGITIDDYNKVSRGMYYYLYR
PlySK1249           NAFNGSAKGTEVLIQSSLEADKEDMAILSLLSRYFQNRGIKKVDWLYNANQAASRGYTYR
PlySs9              NAFNGSAKGTEVLIQSSLEADKEDMAILSLLSRYFQNRGIKKVDWLYNANQAASRGYTYR
EN534               NAFNGTARGTEVLIQSSLTADKEDLAILSVLSRHFQNRGIKKVDWLYNANEAKNRGYTYR
Ply0643             NAFNGQARGSEILIYSGYTADSLDQKLLAILAKRFTNRGFKQVNWLYNANVSASRGYNYR
B30                 GDVAIFMVVDGVTPYSHVAIFDSDAGGGYGWFLGQNQGGANGAYNLVKIPYSATYPTAFR
PlyGBS              GDVAIFMVVDGVTPYSHVAIFDSDAGGGYGWFLGQNQGGANGAYNIVKIPYSATYPTAFR
PlySs2              SSYNWQANGWTVSTSGPFQAGQIVTLGATSGNPYGHVVIVEAVDGDRLTILEQNYGGKRY
                    .              .                          .

PlyCYU              PANQPSISNKSLEQLVKETLAGVHGNGDARKASLGNQYEPVMAVINGKATALK-------
PlySK1249           LVEIAFIDNEQDMAIFETKKEDIAKGLVSAIT-GVEVKTIVPSTPSSTVGSSGTPSKPIY
PlySs9              LVEIAFIDNEQDMAIFENKKEDIAKGLVSAIT-GVEVKTIVPSPPSSTVGSSGTPSKSIY
EN534               LVEIAFIDNEEDMTIFENKKEELAKGLVSAIT-QEEVKTVVSATPSKQGGQPHASTSPVY
Ply0643             LVEIAFIDNNSDVGIYEANKDSMAREFVQAITGQAQVISPSPNTPQSRVTS--------Y
B30                 PKSFKNAVTVTDNTGLNKGDYFIDVSAYQQADLTTTCQQAGTTKTIIKVSES--------
PlyGBS              PKVFKNAVTVTGNIGLNKGDYFIDVSAYQQADLTTTCQQAGTTKTIIKVSES--------
PlySs2              PVRNYYSAAS------------YRQQVVHYITPPGTVAQSAPNLAGS-------------
```

```
PlyCYU          ----KTIDELVQEVIAGKHGNGEERKKSLGSDYDAVQKRVTEILKSDTSGNTPKTPSDTP
PlySK1249       LVGDSLRVLPHATHYQTGQKIANWVKGRTYKILQEKNVHQSNSLRAYLLDGIKSWVLEQD
PlySs9          LVGDSLRVLPHATHYQTGQKIANWVKGRTYKILQVKNVHQSNSKRAYLLDGIKSWVLEQD
EN534           HVGDSVRVLGHATHYQTGQAMASWVKGRTYKILQVKAVNQSRSKRAYLLEGITSWVLEQD
Ply0643         HVGDPVTVQQHATHYQTGQAISSWVKGKTFKVIRVKDVNQSNSKKAYLLEGINSWVLEQD
B30             ---IAWLSDRHQQQANTSDPIGYYHFGRFGGDSALAQREADLFLSNLPSKKVSYLVIDYE
PlyGBS          ---IAWLSDRHQQQANTSDPIGYYHFGRFGGDSALAQREADLFLSNLPSKKVSYLVIDYE
PlySs2          -----RSYRETGTMTVTVDALNVRRAPNTSGEIVAVYKRGESFDYDTVIIDVNGYVWVSY
                                  .                   .

PlyCYU          KSGAVNSSTEPKIEETEPTGNATVNKEEGDLSFNGAILKKAVLDKILANCKKHDILPSYA
PlySK1249       VEGTTKGHSEQTYQAQKGDTYYGIARKFGLTVDALLAVNGLKKTDILRVGQTLKVNAASR
PlySs9          VEGTTKGHSEQTYQAQKGDTYYGIARKFGLSVDTLLVVNGLKKSDILKVGQTLKVNAASR
EN534           VEGTSLGHSEQTYTAQKGDSYWRIARKSGTTVDGLLALNGLKKTDVLKIGQTLKVHKATS
Ply0643         VKGTTNGHSEQTYTVQKGDTLYGIARKFKTSVSELVRLNSIINPSLISVGQKLKLK----
B30             DSASADKQANTNAVIAFMDKIASAGYKPIYYSYKPFTLNNIDYQKIIAKYPNSIWIAGYP
PlyGBS          DSASADKQANTNAVIAFMDKIASAGYKPIYYSYKPFTLNNIDYQKIIAKYPNSIWIAGYP
PlySs2          IGGSGKRNYVATGATKDGKRFGNAWGTFK-------------------------------
                  .:       .

PlyCYU          LTILHYEGLWGTSAVGKADNNWGGMTWTGQGNRPSGVTVTQGTDRPSNEGGHYMHYANVD
PlySK1249       ITTAIPTSVASRVVASALSKVGQKVTVPSNPYGGQCVALVDKIVQELTDKNMSYTNAIDC
PlySs9          TTTAIPTSVASRVVASALSKVGQKVTVPSNPYGGQCVALVDKIVQELTDKNMSYTNAIDC
EN534           SIKAVATSLAQRAVASALSKVGQKVTVPTNPYGGQCVSLVDKIVQELTDKDMAYTNAIDC
Ply0643         ------------------------------------------------------------
B30             DYEVRTEPLWEFFPSMDGVRWWQFTSVGVAGGLDKNIVLLADDSSKMDIPKVDKPQELTF
PlyGBS          DYEVRTEPLWEFFPSMDGVRWWQFTSVGVAGGLDKNIVLLADDSSKMDIPKVDKPQELTF
PlySs2          ------------------------------------------------------------

PlyCYU          DFLTDWFYLLRVGGSYKVSGAKTFSEAVKGMFKVGGAVYDYAASGFDSYIVGASSRLKAI
PlySK1249       LKKAKSNGFQVIYDAWGVNPKAGDFYVIETDGLVYGHIGVCVTDSDGKSIDGVEQNIDGY
PlySs9          LKKAKSNGFQVIYDAWGVNPKAGDFYVIQTDGMVYGHIGVCVTDSDGKSIDGVEQNIDGY
EN534           LTKAKANGFTVIKDAWGVNPKAGDFYVIKTDSHPYGHIGICITDSDGTSIDGVEQNVDGY
Ply0643         ------------------------------------------------------------
B30             YQKLATNTKLDNSNVP---------YYEATLSTDYYVESKPNASSADKEFIKAGTRVRVY
PlyGBS          YQKLATNTKLDNSNVP---------YYEATLSTDYYVESKPNASSADKEFIKAGTRVRVY
PlySs2          ------------------------------------------------------------

PlyCYU          ESENGSLDKFDKQTVSDVVQSDNIEINVEGIEVIINGETYRLEKKPV-----
PlySK1249       SDHNKNGINDQLEIGGGGITRRVKRQWMADGSLYDSTGTVKLGKVVGWFRIS
PlySs9          SDHNNNGINDQLEIGGGGITRRVKRQWMANGSLYDSTGTVKLGKVVGWFRIS
EN534           SDHNKNGINDQLEIGGGGITRRVKRVWMADGSLYDATGTVKLGKVIGWFRLG
Ply0643         ----------------------------------------------------
B30             EKVN----------GWSRINHPESAQWVEDNYLVNATDM-------------
PlyGBS          EKVN----------GWSRINHPESAQWVEDSYLVNATDM-------------
PlySs2          ----------------------------------------------------
```

Fig. S3 Antibacterial activities by turbidity reduction assay of PlyCYU-Cys34Ala, PlyCYU-Cys34Ser, and PlyCYU-His99Ala (50 µM) in comparison to PlyCYU (2.5 µM) against *S. agalactiae* serotype II. Reaction without endolysin (marked as no endolysin with blue dot line) was included as a control. Black and grey lines represent reactions carried out in the presence of PlyCYU and its indicated variants, respectively.

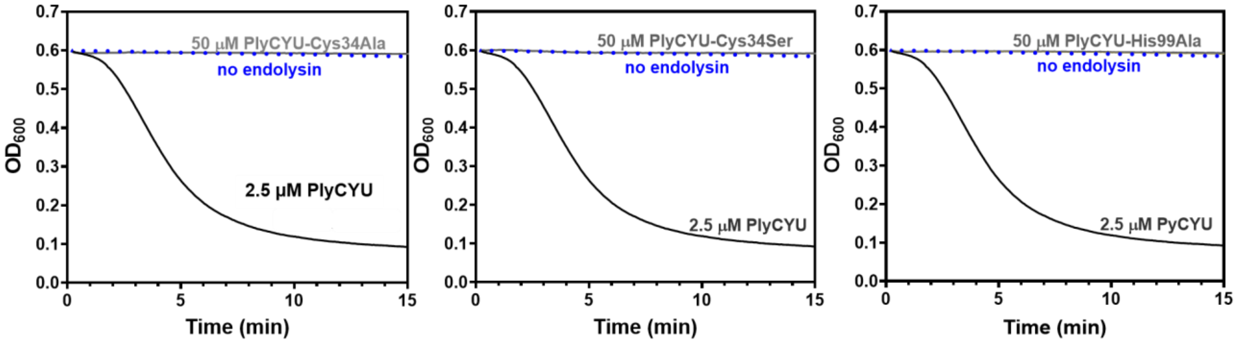

Fig. S4 Analyses of molecular mass and oligomeric state of PlyCYU and variants using (I) SEC-MALS and (II) traditional SEC-UV.

(I) SEC-MALS chromatograms of (a) PlyCYU, (b) PlyCYU277, (c) cyuLyz2 and (d) CW7_Lyz2. The average molecular masses (kDa) are from the corresponding molecular weight (MW) distributions shown as blue horizontal lines in the elution peaks.

(II) SEC-UV. (a) Chromatograms of protein molecular mass standards−thyroglobulin (T; 669 kDa), ferritin (F; 440 kDa), aldolase (AL; 158 kDa), conalbumin (Con; 75 kDa), ovalbumin (O; 43 kDa), carbonic anhydrase (CA; 29 kDa), ribonuclease (R; 13.7 kDa) and aprotinin (AP; 6.5 kDa)−and blue dextran (B; grey line). (b) A calibration curve of the relative volume ratios (V_e/V_o) *versus* the logarithms MW of standard proteins. Standard proteins are in black circles, whereas PlyCYU variants are labeled and denoted by the colored squares. (c) chromatograms of PlyCYU (black; 368 kDa for heptamer), CW7_Lyz2 (brown; 447 kDa for dodecamer), cyuLyz2 (blue; 176 kDa for heptamer), PlyCYU277 (green; 28 kDa for monomer), and PlyCYU214 (purple; 8 kDa for monomer). (d) Chromatograms of PlyCYU214 performing at 150 (pink) and 500 mM (grey) NaCl, respectively.

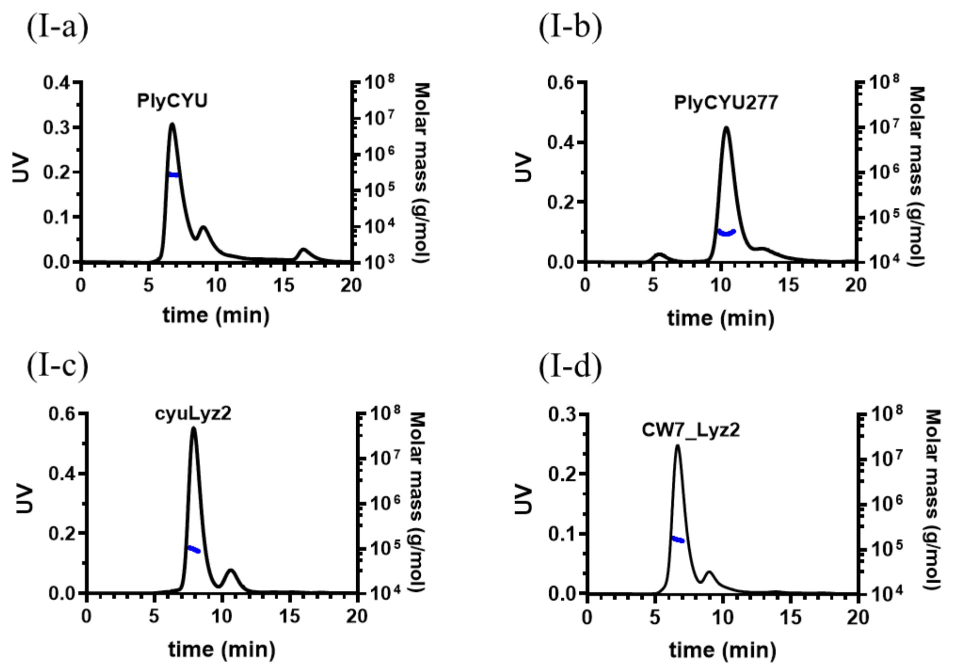

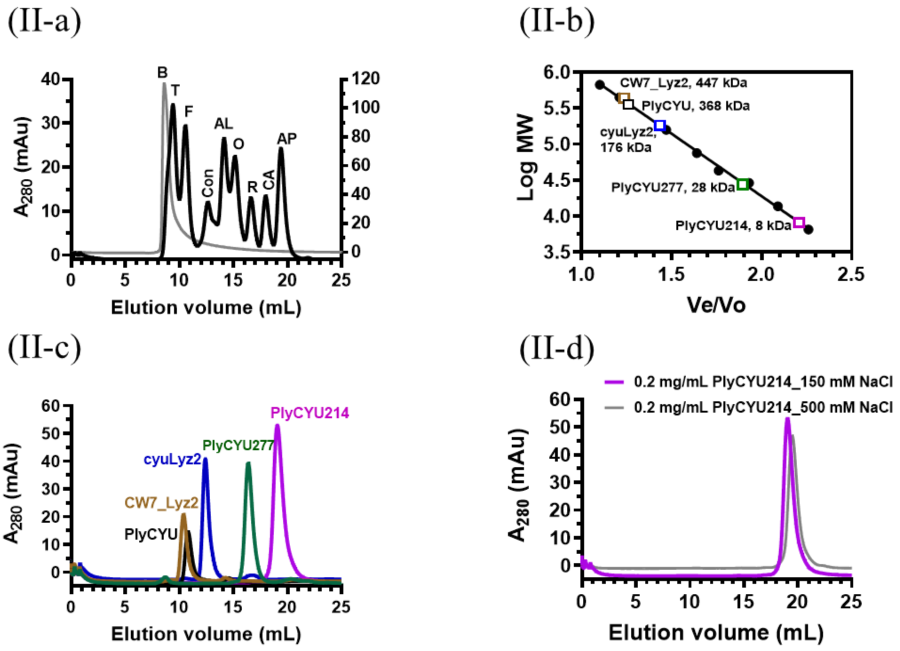
(II-a)
A280 (mAu)
B
T
F
Con
AL
O
R
CA
AP
Elution volume (mL)
(II-b)
Log MW
CW7_Lyz2, 447 kDa
PlyCYU, 368 kDa
cyuLyz2, 176 kDa
PlyCYU277, 28 kDa
PlyCYU214, 8 kDa
Ve/Vo
(II-c)
A280 (mAu)
PlyCYU214
PlyCYU277
cyuLyz2
CW7_Lyz2
PlyCYU
Elution volume (mL)
(II-d)
0.2 mg/mL PlyCYU214_150 mM NaCl
0.2 mg/mL PlyCYU214_500 mM NaCl
A280 (mAu)
Elution volume (mL)
